# Accelerated epigenetic age is associated with whole-brain functional connectivity and impaired cognitive performance in older adults

**DOI:** 10.1038/s41598-024-60311-3

**Published:** 2024-04-26

**Authors:** Andrew J. Graves, Joshua S. Danoff, Minah Kim, Samantha R. Brindley, Amalia M. Skyberg, Stephanie N. Giamberardino, Morgan E. Lynch, Brenda C. Straka, Travis S. Lillard, Simon G. Gregory, Jessica J. Connelly, James P. Morris

**Affiliations:** 1 University of Virginia, Charlottesville, USA; 2 University of Oregon, Eugene, USA; 3 Duke University, Durham, USA; 4 University of Southern California, Los Angeles, USA; 5 University of Illinois Urbana-Champaign, Urbana, USA

**Keywords:** Cognitive ageing, Cognitive neuroscience, Epigenetics and behaviour, Psychology

## Abstract

While chronological age is a strong predictor for health-related risk factors, it is an incomplete metric that fails to fully characterize the unique aging process of individuals with different genetic makeup, neurodevelopment, and environmental experiences. Recent advances in epigenomic array technologies have made it possible to generate DNA methylation-based biomarkers of biological aging, which may be useful in predicting a myriad of cognitive abilities and functional brain network organization across older individuals. It is currently unclear which cognitive domains are negatively correlated with epigenetic age above and beyond chronological age, and it is unknown if functional brain organization is an important mechanism for explaining these associations. In this study, individuals with accelerated epigenetic age (i.e. AgeAccelGrim) performed worse on tasks that spanned a wide variety of cognitive faculties including both fluid and crystallized intelligence (*N* = 103, average age = 68.98 years, 73 females, 30 males). Additionally, fMRI connectome-based predictive models suggested a mediating mechanism of functional connectivity on epigenetic age acceleration-cognition associations primarily in medial temporal lobe and limbic structures. This research highlights the important role of epigenetic aging processes on the development and maintenance of healthy cognitive capacities and function of the aging brain.

## Introduction

The body and mind undergo significant changes as we age through the lifespan^1,2^. These developmental trajectories are critical for healthy development, reducing risk of mortality, and overall well-being^3–5^. While chronological age is a strong predictor for health-related risk factors, it is an incomplete metric that fails to fully characterize the unique aging process of individuals with different genetic makeup, neurodevelopment, and environmental experiences^6,7^. One of the most striking changes humans undergo as we age is general decline across a wide variety of cognitive faculties, including memory, reasoning, spatial visualization, and processing speed^8^. The current research is focused on taking an interdisciplinary approach for understanding cognitive aging through an epigenetic and neuroimaging framework.

Recent advances in epigenomic array technologies have made it possible to generate DNA methylation-based biomarkers of biological aging^9^. These biomarkers, called epigenetic clocks, use DNA methylation values from CpG sites across the genome to estimate the biological age of a person or tissue. One major advantage of epigenetic clocks is that they can be measured from all sources of DNA, including peripheral tissues, and can be applied throughout the lifespan^10^. The first epigenetic clock, Horvath’s multi-tissue age estimator, yields epigenetic age estimates highly correlated with chronological age^10^. Biological aging can be differentiated from chronological age by taking the residuals leftover from the linear relationship between epigenetic age and chronological age, which captures the information embedded in the biomarker unexplained by chronological age. A higher epigenetic age compared to an individual’s chronological age would indicate they are aging faster than expected. This epigenetic age acceleration is associated with risk of mortality and various age-related diseases including cancer, cardiovascular diseases, and dementia^11^. Additionally, it is possible to assess these biomarkers of aging prior to the onset of disease^12,13^. After the publication of Horvath’s original multi-tissue age estimator (i.e. first-generation clocks: acceleration denoted as AgeAccelerationResidual), many other epigenetic clocks have been developed that capture different aspects of aging and are sensitive to external environmental factors (i.e. second-generation clocks)^7^. One second-generation clock is DNAmPhenoAge (acceleration denoted as AgeAccelPheno), which is trained on “phenotypic age” instead of chronological age. The phenotypic age is itself an estimator of biological age based on physiological measurements that are associated with increased mortality^14^. The current research is utilizing DNAmGrimAge, an epigenetic clock that was developed to reflect physiological changes associated with aging, including known plasma protein biomarkers of aging, and is trained on time-to-death^15^. DNAmGrimAge age acceleration (denoted as AgeAccelGrim) stands out among epigenetic clocks in its capacity to predict mortality risk and clinically relevant measures of aging^15,16^.

While most research has focused on clinically relevant outcomes related to epigenetic age, several studies have investigated the link between epigenetic aging processes and cognitive abilities^17–20^. One study found that age-adjusted Horvath’s clock values negatively correlated with a single *g*-factor derived from cognitive items using principal components analysis in the Lothian Birth cohort^21^. A similar finding was reported in another sample, and found that digit symbol substitution, symbol search 4-choice reaction time, and matrix reasoning survived inference criterion after mass-univariate hypothesis testing from a collection of cognitive items^22^. A longitudinal twin-study found that twins with higher epigenetic age acceleration relative to their twin sibling experienced more cognitive decline, which directly points to a role of epigenetic modification in cognitive aging^23^. However, a different twin-study using the original Horvath and Hannum methods did not find evidence for a link between epigenetic age acceleration and cognition^24^.

A qualitative meta-analysis of the current literature, which includes a heterogenous collection of epigenetic clock and cognitive measurements, suggests that the link between epigenetic age acceleration and cognition is potentially promising but currently unclear and inconsistent^25^. The pattern of results in the literature is heavily dependent upon the epigenetic clock chosen as well as the investigated cognitive domain, with second-generation epigenetic clocks typically outperforming first-generation epigenetic clocks for detecting associations^26^. And while studies exist that have looked at structural brain changes such as BrainAge estimators, there is a dearth of research examining functional brain changes with functional magnetic resonance imaging (fMRI) for age accelerated individuals^27^. This is important because differences in functional brain organization is a putative mechanism for explaining the potential link between epigenetic age and cognition^28^.

With respect to individual differences in human brain activity, the aging process has been examined with resting-state functional connectivity (rsFC) in fMRI. Resting-state functional networks measure the temporal co-activation of low frequency blood-oxygen-level-dependent (BOLD) signals when individuals are “at rest” and have shown to be stable across timepoints^29,30^. Studies on healthy adults have consistently documented aging-related decreases in long-range connectivity within the Default Mode Network (DMN), comprised of the medial prefrontal cortex, the posterior cingulate cortices, hippocampus and the inferior parietal lobules^31–35^. These declines in connectivity were preserved after controlling for structural gray matter volume^33,36^. In addition, aging has been associated with decreased connectivity in rsFC networks associated with attention, salience and/or motor regions^36–38^ and increased connectivity in networks related to sensorimotor and subcortical structures^38^.

Variability in rsFC has also been found to map onto individual differences in cognitive abilities^39,40^. In aging individuals, increased connectivity in the DMN have been related to better scores on a memory task^41^, a cognitive control task^42^, a motor speed task^43^, and an executive functioning and processing speed task^33^. However increased connectivity does not always associate with better cognitive performance in this age group: Decreased interhemispheric coupling of language processing areas were found to be positively correlated with grammar learning^44^ and decreased connectivity between the thalamus and basal ganglia were positively related with verbal episodic memory^45^. Newer studies have cited interhemispheric connectivity and connectivity within the cingulo-opercular network as important neural correlates of cognitive skills in aging adults^46,47^. In summary, rsFC has utility in helping us understand age-related changes and variability in cognitive aging.

One particularly influential analysis approach for understanding individual differences in resting-state brain networks is the connectome-based predictive model (CPM)^48^. CPM leverages robust idiosyncrasies of functional connectivity across individuals to make phenotypic predictions. CPM is particularly suited for individual differences research, as the networks are highly predictive of an individual’s identity irrespective of nuisance artifacts such as head motion or anatomical differences^49^. Successful analysis frameworks using CPM include predicting an individual’s ID, age, attention, and general intelligence, among other phenotypes^48,50,51^. Because epigenetic age acceleration and chronological age capture unique components of the aging process, it is currently unclear due to the dearth of research whether or not epigenetic age acceleration is related to individual differences in functional brain connectivity. To our knowledge, no research group has published work on using rsFC to examine changes in AgeAccelGrim.

The approach for the current research is unique because we are investigating an entire cognitive battery and decomposing those items into interpretable latent factors using a network model, and then using neuroimaging as a tool to understand how these relationships may be represented in the brain. This is in contrast to the previous studies investigating the relationship between epigenetic age and cognition, which have either (1) performed principal components analysis to estimate a single *g*-factor based on only one principal component, (2) only analyzed statistical relationships at the item-level which may be limited by the idiosyncratic properties of the task relevant to that item, and (3) importantly, did not use fMRI to build connectome-based predictive models to characterize these relationships in the brain. In other words, it is unclear which cognitive domains are consistently negatively correlated with epigenetic age acceleration, and it is unknown if functional brain organization is an important mechanism for explaining these associations. We are also including measures of crystallized intelligence (e.g. vocabulary assessments), in order to disambiguate if the relationship between epigenetic age processes and cognition is driven solely by fluid intelligence constructs (i.e. processing speed, spatial reasoning, etc.) or not^52^. This is an important distinction, given that crystallized intelligence typically does not decline with age and may not be as sensitive to the aging brain to the same degree as fluid intelligence, which will inform the scope of the effect of epigenetic processes on healthy cognition and brain function^53^.

The first goal of the current research is to test whether epigenetic aging processes (as indexed by AgeAccelGrim) predict individual differences between people across multiple cognitive ability domains. We chose to look at AgeAccelGrim, because empirical evidence suggests that this clock is more consistently associated with cognitive abilities relative to other epigenetic clocks^25^. Specifically, we hypothesize that individuals who are more age accelerated with respect to their epigenome will perform worse on a wide variety of cognitive assessments that measure various facets of both fluid and crystallized intelligence. To answer this question, we used a subset of the Virginia Cognitive Aging Project (VCAP) cohort who provided samples for epigenetic analysis and underwent fMRI. VCAP is one of the world’s largest longitudinal studies of cognitive change with a sample of community-dwelling healthy older adults, and has recently been enriched by data collection protocols incorporating epigenomic and neuroimaging data^54^. This subset of VCAP participants includes individuals with scores showing signs of cognitive decline and individuals with scores showing signs of cognitive improvement (estimated by their previous visits). To index cognitive performance across a wide variety of domains, we used the VCAP cognitive battery, which includes 15 scales that map onto the latent domains of processing speed, memory, spatial visualization, reasoning, and vocabulary (three scales for each domain). This will help clarify whether or not second-generation epigenetic clocks, such as AgeAccelGrim, do in fact negatively correlate with cognition.

The second goal of the current research is to test whether AgeAccelGrim can be predicted from an individual’s rsFC fMRI profile using ridge regression connectome-based predictive models (rCPM). We are specifically interested in whether or not individual differences in cognition account for the potential relationship between rsFC and AgeAccelGrim. If functional networks were identified as being predictive, we used graph-theoretic tools to estimate which brain regions are important for contributing to overall model performance. We hypothesize that functional connectivity of brain structures that can predict AgeAccelGrim are important functional hubs for higher-order cognitive abilities such as those measured by the VCAP cognitive battery. We aimed to determine whether the functional connectivity profiles are reflective of general cognitive abilities (i.e. *g*-factor), or a more specific process such as processing speed or memory. Answering these questions will enrich our understanding of if and how epigenetic age acceleration relates to functional brain network connectivity in the context of cognitive differences across individuals. Furthermore, finding evidence in functional brain networks may help explain why there is a link between epigenetic aging and cognitive aging processes.

## Results

### Marginal correlations between AgeAccelGrim and all measured cognitive items are negative

**Figure 1:**
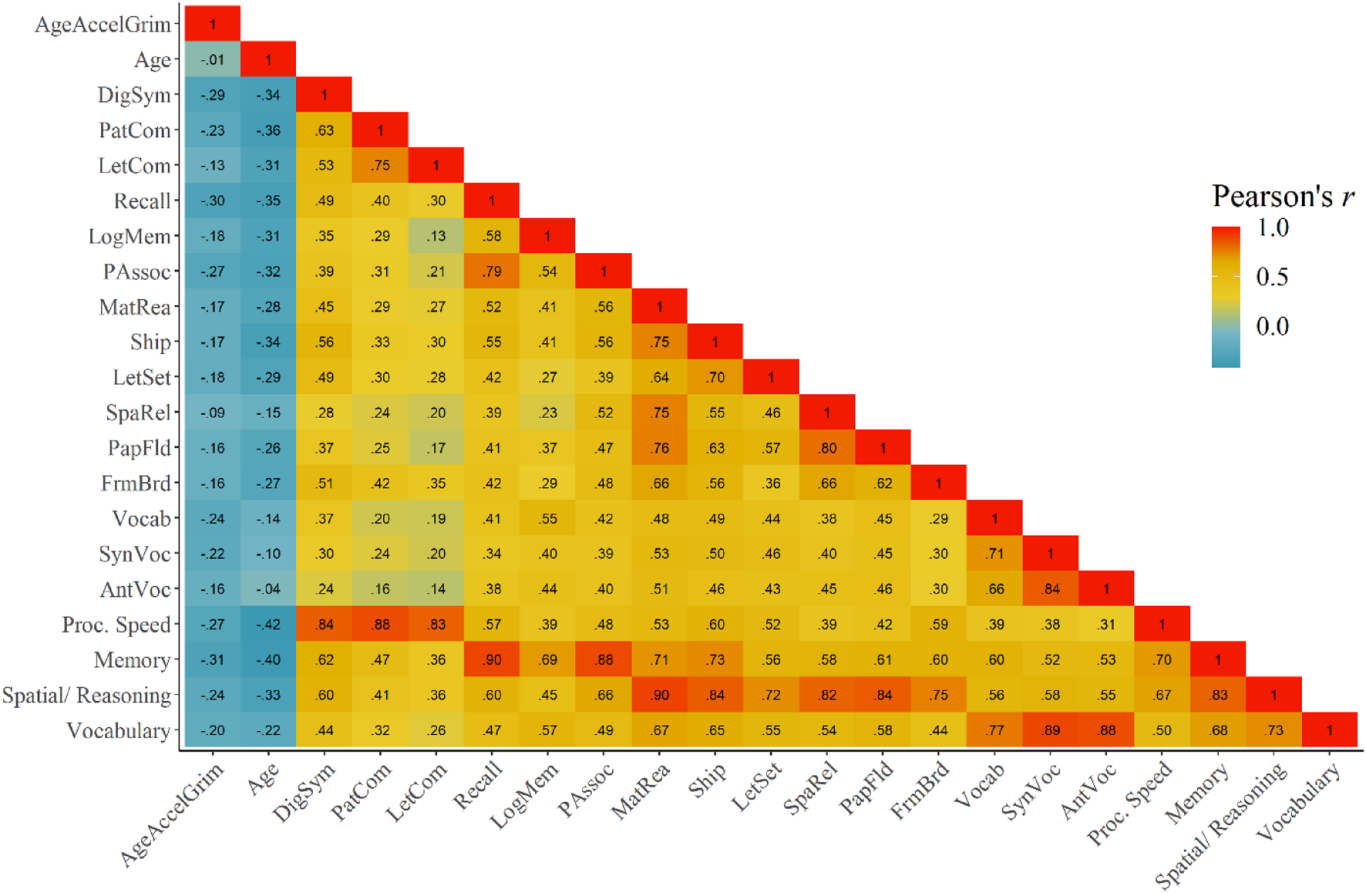
AgeAccelGrim negatively correlates with all cognitive items measured. Shown is the raw correlation matrix between AgeAccelGrim, chronological age, the original cognitive items, and the 4 latent cognitive domains. Data is aggregated over the three sessions by taking the mean for this visualization. There is no linear relationship between AgeAccelGrim and chronological age. At the marginal level, both AgeAccelGrim and chronological age negatively associate to some degree with all of the cognitive items surveyed, as well as the 4 latent domains. As expected, the cognitive items and latent cognitive domains are positively correlated with each other.

In order to directly compare results from the item-level cognitive data to the latent-level cognitive data, we computed a simple Pearson’s correlation matrix between all cognitive items, latent variables, AgeAccelGrim, and chronological age (see Fig. 1). Participant level summary statistics for cognitive performance and demographics are included in Table 1. For this analysis, we are not making any conclusions based on significance testing, but rather describing the pattern of marginal associations between all of these variables before fitting statistical models. For this specific analysis, data are aggregated over session by taking the mean cognitive score across sessions. There are several important pieces of information to glean from this analysis. First, by design, there is no linear relationship between AgeAccelGrim and chronological age; these two random variables are orthogonal with each other. Second, the sign of the marginal correlation between AgeAccelGrim and all cognitive items are negative. As expected, the same is true for chronological age. Third, the correlation between all cognitive items are positive, exhibiting the classical pattern of a positive manifold in cognitive testing^55^. Fourth, the latent variables estimated from bootEGA (denoted as Proc. speed, memory, spatial/reasoning, and vocabulary) capture the unique contributions of the items that belong in their respective communities, by exhibiting larger positive correlations within-community relative to between-community. Finally, because the latent variables themselves exhibit a degree of positive correlation, this motivates the use of a multivariate statistical model to account for the linear relationships between the response variables.

**Table 1:** VCAP participant summary statistics.

Trait	Minimum	Mean	Maximum	SD
Age-related variables				
Age	59	68.98	81	5.68
AgeAccelGrim	− 6.46	− 0.16	9.88	3.49
DNAmGrimAge	54.77	64.95	79.84	5.52
Time difference (days)	21	331.54	1288	124.13
Cognitive measurements				
DigSym	35	72.24	107.67	12.61
PatCom	6.5	14.78	25.5	3.2
LetCom	4.5	9.71	17.17	2.05
Recall	17.33	34.32	46.67	6.2
LogMem	21.67	48.33	64	8.32
PAssoc	0.33	2.79	5.83	1.51
MatRea	2	7.26	13.33	2.81
Ship	4	12.86	18.67	2.91
LetSet	3.33	11.36	14	1.87
SpaRel	3.33	9.49	16.67	3.43
PapFld	2	6.16	11	2.44
FrmBrd	1	6.43	13	2.8
Vocab	17.67	53.59	62.67	6.87
SynVoc	3.33	7.82	9.67	1.47
AntVoc	2.67	7.35	10	1.65

Cognitive measurements were averaged across the three sessions here for computing participant-level summary statistics.

### AgeAccelGrim negatively associates with cognitive performance conditioning on chronological age and relevant covariates

**Figure 2:**
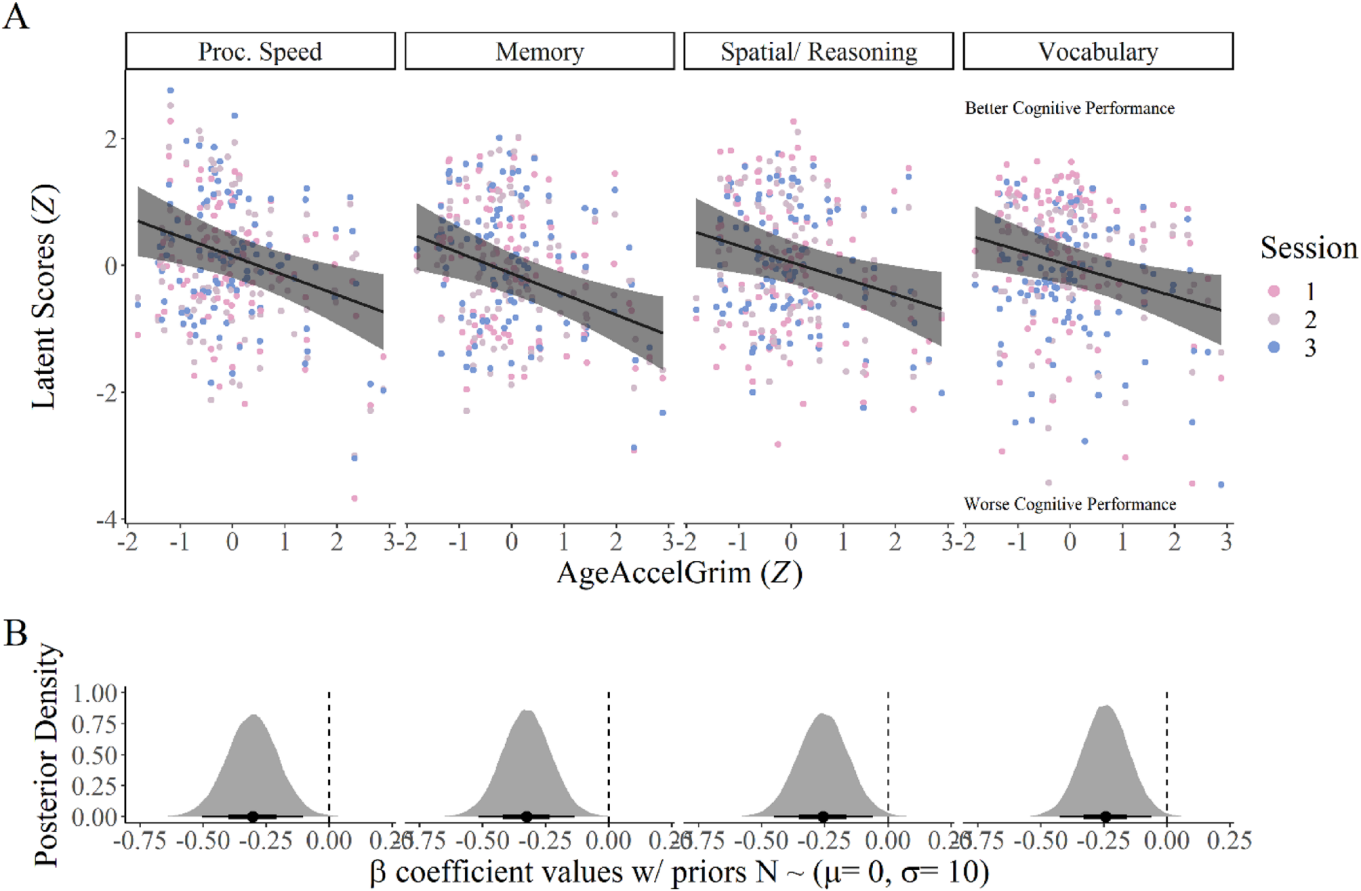
AgeAccelGrim negatively associates with all 4 latent cognitive domains after adjusting for chronological age and all covariates. Panel (**A**) shows the posterior estimates for the AgeAccelGrim regression coefficients for each response variable as black lines with uncertainty bands showing the uncertainty. Overlaid are the raw data used to fit the model, which includes session-level information. Panel (**B**) shows the density plots on the bottom of the entire posterior distribution of parameter values, with 95% credible intervals indicated by the lines underneath the density. There is very little overlap with 0, suggesting that the relationship between AgeAccelGrim, conditional on chronological age and all other covariates, is reliably negative. The actual posterior probability values are included in the main text.

Results from the multivariate hierarchical Bayesian model indicate that AgeAccelGrim does in fact negatively correlate with cognitive performance across all four empirically derived latent domains (see Fig. 2 and Table 2). This finding suggests that accelerated epigenetic age is associated with between-person differences in worse cognitive performance across tasks that measure both fluid and crystallized intelligence. Using the analogous Bayesian procedure for computing adjusted probability values directly from the posterior distributions on the standardized regression coefficients, processing speed (β = − 0.31, *p* = 0.007), memory (β = − 0.33, *p* = 0.004), reasoning/ spatial visualization (β = − 0.26, *p* = 0.009), and vocabulary (β = − 0.24, *p* = 0.009) all survive our inference threshold of α = 0.05 using the False Discovery Rate (FDR) procedure for multiple comparisons (see Fig. S1 for posterior fits)^56^. This further corroborates with the evidence from Fig. 1 that demonstrates the marginal correlation between AgeAccelGrim and every single cognitive survey administered has a negative sign. Results from the model provide further evidence for a direct relationship between AgeAccelGrim and cognitive performance by conditioning on chronological age, sex, time factors, and blood cell count indices. By design, AgeAccelGrim and chronological age are fully orthogonal to each other, which suggests that AgeAccelGrim explains a unique portion of the variance in the response variable not captured by chronological age, because both covariates are included in the model estimation. As expected, chronological age was also negatively related to all four cognitive domains. The standardized AgeAccelGrim β coefficient maximum a-posterior (MAP) estimates for all four cognitive variables fall between − 0.24 and − 0.33. This suggests that a one standard deviation increase in AgeAccelGrim results in approximately one-fourth/one-third of a standard deviation decrease across all measured cognitive faculties.

**Table 2:** Bayesian multivariate population parameter estimates and associated uncertainty.

β coefficient	Proc. speed	Memory	Spatial/reasoning	Vocabulary
Parameters of interest				
Intercept	0.15 [− 0.19, 0.49]	− 0.13 [− 0.46, 0.20]	0.05 [− 0.28, 0.39]	0.00 [− 0.31, 0.31]
** AgeAccelGrim**	− **0.31 [**− **0.51,** − **0.11]**	− **0.33 [**− **0.52,** − **0.13]**	− **0.26 [− 0.45,** − **0.06]**	− **0.24 [− 0.43,** − **0.06]**
** Age**	− **0.39 [**− **0.58,** − **0.21]**	− **0.45 [**− **0.62,** − **0.27]**	− **0.33 [− 0.51,** − **0.15]**	− **0.20 [**− **0.37,** − **0.04]**
Covariates				
Time difference	0.03 [− 0.14, 0.20]	0.10 [− 0.06, 0.27]	0.14 [− 0.03, 0.30]	0.01 [− 0.14, 0.17]
Session	**0.06 [0.02, 0.10]**	0.01 [− 0.03, 0.04]	0.02 [− 0.02, 0.07]	** − 0.18 [− 0.23, − 0.12]**
Sex (female)	− 0.21 [− 0.63, 0.21]	0.18 [− 0.22, 0.58]	− 0.06 [− 0.47, 0.35]	0.09 [− 0.28, 0.47]
NK	0.05 [− 0.22, 0.32]	0.18 [− 0.08, 0.43]	0.17 [− 0.09, 0.43]	0.07 [− 0.17, 0.30]
Mono	− 0.11 [− 0.30, 0.07]	− 0.10 [− 0.28, 0.08]	− 0.11 [− 0.29, 0.07]	− 0.06 [− 0.22, 0.11]
Gran	− 0.05 [− 0.41, 0.30]	0.03 [− 0.31, 0.36]	0.04 [− 0.31, 0.38]	0.14 [− 0.18, 0.45]
PlasmaBlast	0.19 [− 0.07, 0.44]	0.11 [− 0.13, 0.35]	0.06 [− 0.19, 0.30]	0.01 [− 0.21, 0.24]
CD8pCD28nCD45RAn	− 0.02 [− 0.24, 0.21]	0.02 [− 0.19, 0.23]	0.09 [− 0.12, 0.31]	0.06 [− 0.13, 0.27]
CD8.naive	0.11 [− 0.14, 0.35]	0.03 [− 0.21, 0.26]	0.02 [− 0.22, 0.25]	0.09 [− 0.14, 0.31]
CD4.naive	− 0.12 [− 0.38, 0.14]	− 0.10 [− 0.35, 0.15]	− 0.04 [− 0.29, 0.22]	− 0.17 [− 0.40, 0.06]

Bold cell entries indicate 95% credible intervals that do not overlap with 0.

### Both AgeAccelGrim and age can be predicted from functional connectomes, and these results are accounted for via individual differences in cognition

**Figure 3:**
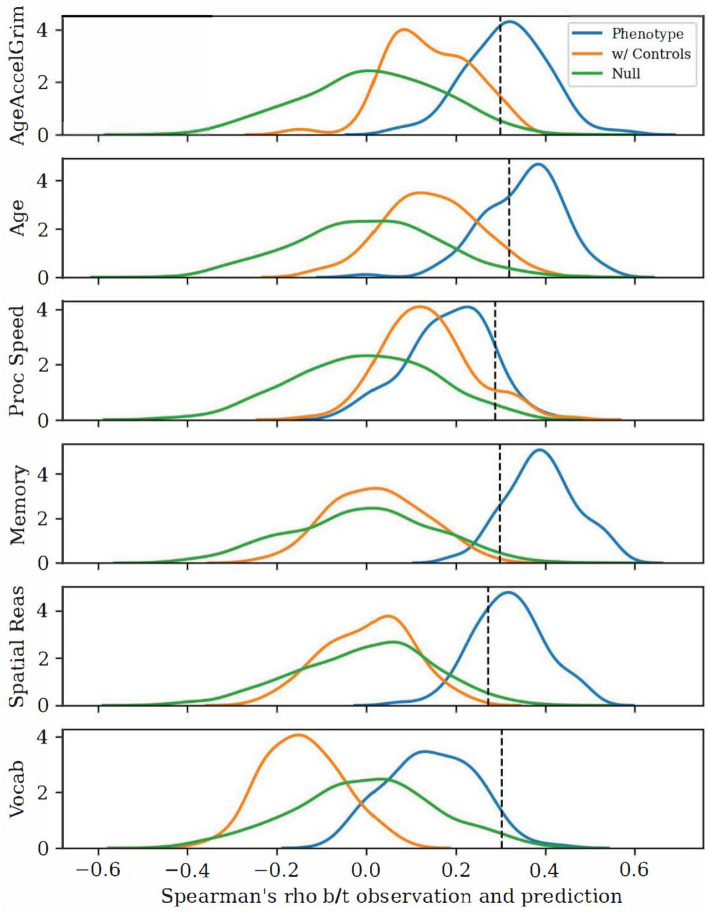
rCPM significantly predicts AgeAccelGrim, age, and memory/ spatial reasoning. Using both fMRI resting state input scans, rCPM can predict epigenetic age and chronological age, as well as memory and spatial reasoning (which are highly positively correlated). The phenotype (blue) distributions for the AgeAccelGrim analysis controlled for age, sex and the time difference between blood draw/brain scan and cognitive assessment, and conversely the age analysis controlled for AgeAccelGrim, sex, and the time difference. The control-adjusted (orange) distributions for AgeAccelGrim and Age additionally controlled for all four cognitive factors. The phenotype (blue) distributions of the cognitive factors controlled for sex and time difference. The control-adjusted (orange) distributions for all four cognitive factors additionally controlled for Age and AgeAccelGrim. These results show that the functional connectome contains information uniquely relevant for both epigenetic age and chronological age, and that information is shared with individual differences in cognition, particularly memory.

Using rCPM, we are able to significantly predict AgeAccelGrim (median Spearman’s ρ = 0.313, non-parametric *p* = 0.021) and chronological age (median Spearman’s ρ = 0.364, non-parametric *p* = 0.012) from resting-state functional connectivity. Figure 3 shows the distributions of model performance, and suggests that controlling for cognition mitigates the ability to predict AgeAccelGrim and age, suggesting that functional connectivity in the brain is a potential mediating mechanism that explains the relationship between AgeAccelGrim and cognition. In particular, it seems to be the case that memory (median Spearman’s ρ = 0.391, non-parametric *p* = 0.005) and spatial visualization/ reasoning (median Spearman’s ρ = 0.316, non-parametric *p* = 0.012) are able to be predicted from functional connectomes and share the most information with epigenetic age and chronological age-functional connectivity associations. In contrast, processing speed (median Spearman’s ρ = 0.194, non-parametric *p* = 0.101) and vocabulary abilities (median Spearman’s ρ = 0.138, non-parametric *p* = 0.183) were not able to be predicted from resting-state functional connectomes. Figure S2 shows example representations computed when fitting rCPM models, which are highly correlated with simple bivariate correlations between pairwise connectivity and AgeAccelGrim, and suggest interpretability of the resulting coefficients.

### The most important brain regions within the connectome for predicting AgeAccelGrim and Age support memory processes

**Figure 4:**
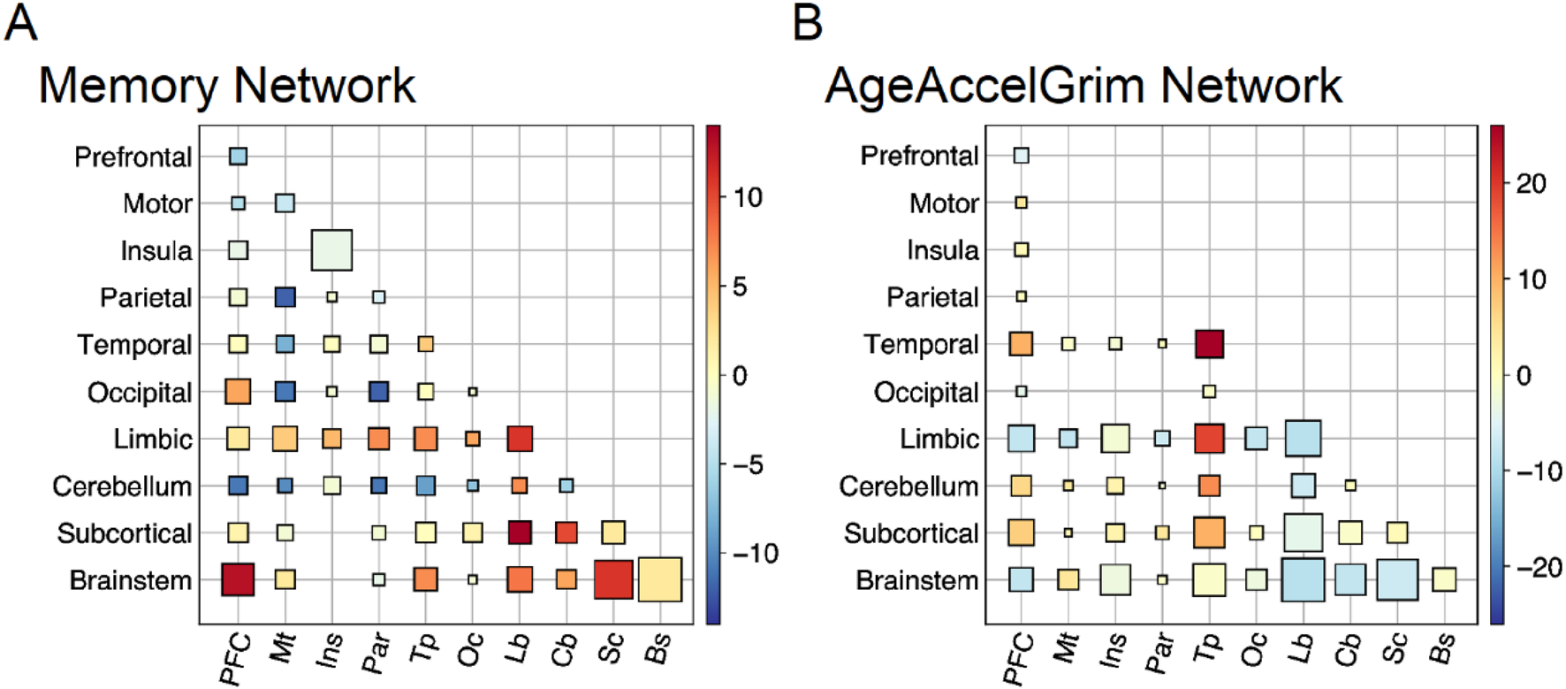
Networks reflective of memory performance and AgeAccelGrim show largely opposite patterns at the lobe-level. Hinton plot visualizations show that memory and AgeAccelGrim have opposing network structure patterns, suggesting that better memory and less accelerated epigenetic age (and vice versa) are captured through similar lobe-level connections through the functional connectome. Size corresponds to the sum of edges in the “high”- and “low” networks standardized by the number of possible edges between each pair of regions. Color corresponds to the difference between edges in the high- and low-phenotype networks, such that red corresponds to edges mostly in the better memory/more epigenetic age accelerated network and blue corresponds to edges mostly in the worse memory/less epigenetic age accelerated network. In particular, the limbic, cerebellar, and brainstem networks, as well as the subcortical connections to those three networks, have opposing strength. These results are in line with the hypothesis that epigenetic age acceleration and memory performance are negatively correlated at least in part due to functional brain activity differences.

**Figure 5:**
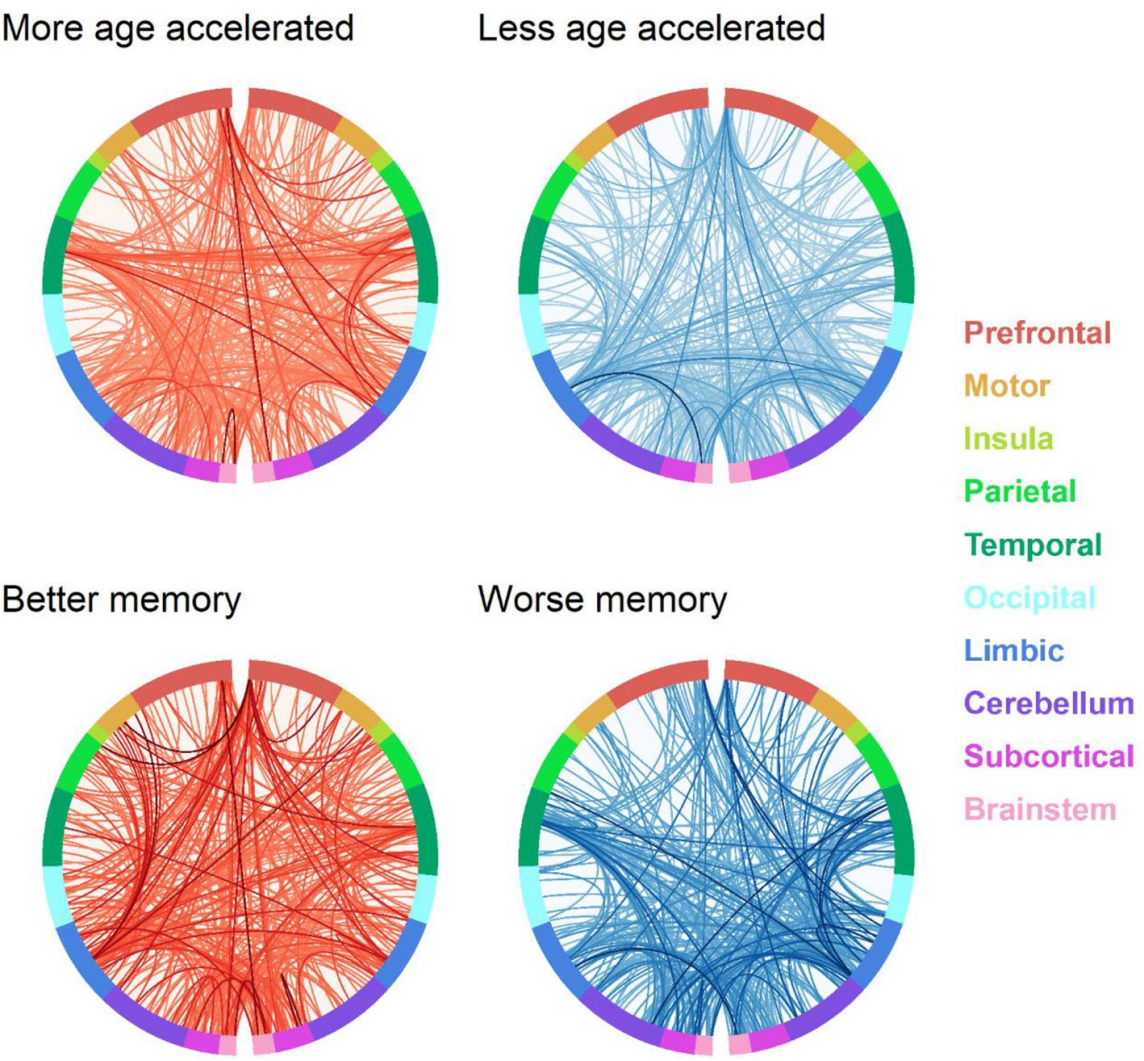
Networks reflective of memory performance and AgeAccelGrim show largely opposite patterns at the edge-level. Circle plot diagrams show that memory and AgeAccelGrim have opposing network structure patterns, suggesting that better memory and less accelerated epigenetic age (and vice versa) are captured through similar edge-level connections through the functional connectome. Edges in the positive networks are visualized in red, and edges in the negative network are visualized in blue. Darker lines correspond to edges with higher strength (absolute value). The hemispheres are split vertically in each panel and are displayed from right to left.

Using eigenvector centrality to estimate which brain regions are important within the rCPM model, we found that the top five brain regions used to predict AgeAccelGrim and memory are similar, and that these structures largely support memory processes. Table 3 shows the most important brain regions in terms of functional connectivity that support a healthy aging process indexed by less epigenetic age acceleration and better memory performance. These brain regions include medial temporal lobe structures such as the hippocampus, parahippocampal cortex, anterior temporal lobe, orbitofrontal cortex, and retrosplenial cortex. Table 4 shows the most important brain regions that indicate a more at-risk aging process indexed by more epigenetic age acceleration and worse memory performance. Interestingly, this model returned some similar nearby patches of subcortex including hippocampal regions within the medial temporal lobe, as well as the caudate nucleus and amygdala. Because of the similarity of results across both the positive and negative weight models, particularly for the medial temporal lobes, it appears that more functional connectivity in some medial temporal lobe structures is indicative of healthy aging while in others less functional connectivity. Figures 4 and 5 show that networks for more epigenetic age acceleration and worse memory (and vice versa) have similar structure, as indexed by opposing color patterns particularly in the limbic system, but also in cerebellum, brainstem, and subcortical connections to proximal lobes. Fig. S3 shows that this structure is specific to AgeAccelGrim, by demonstrating that chronological age does not show the same opposing color pattern in the Hinton diagrams with respect to the memory networks. Table S1 includes the most important regions for predicting older age and younger age, which also include hippocampus, parahippocampus, and orbitofrontal cortex, as well as the caudate. Importantly, the important brain regions detected with AgeAccelGrim and age are different nodes in the network, thus adding unique information and complementary information when considered together.

**Table 3:** Most important functional connectivity regions for better memory and less accelerated epigenetic age.

Node #	X	Y	Z	Region	Lobe	Centrality	Top neurosynth terms
Better memory							
** 94**	**35.6**	** − 14.7**	** − 18.4**	**Hippocampus**	**Limbic**	**0.17**	**Hippocampus, hippocampal, memory**
** 136**	** − 5.8**	**18.2**	** − 21.6**	**Orbitofrontal**	**Prefrontal**	**0.16**	**Subgenual, major depression, depression**
195	− 37.8	− 13.2	− 29.3	Inferior temporal gyrus	Motor	0.14	Anterior temporal, temporal, medial temporal
18	26.6	19.6	− 21.3	Pars orbitalis	Prefrontal	0.13	Cortex ofc, ofc, orbitofrontal
3	5.1	34.9	− 17.4	Orbitofrontal	Prefrontal	0.12	Orbitofrontal, orbitofrontal cortex, medial orbitofrontal
125	14	8.3	− 9.5	Putamen	Prefrontal	0.12	Reward, ventral striatum, striatum
Less age accelerated							
** 94**	**35.6**	** − 14.7**	** − 18.4**	**Hippocampus**	**Limbic**	**0.20**	**Hippocampus, hippocampal, memory**
** 136**	** − 5.8**	**18.2**	** − 21.6**	**Orbitofrontal**	**Prefrontal**	**0.15**	**Subgenual, major depression, depression**
268	− 6.1	− 18.9	− 36.8	Brainstem	Brainstem	0.14	N/A
227	− 7.5	− 42.1	13.3	Agranular retrolimbic	Limbic	0.13	Retrosplenial cortex, retrosplenial, posterior cingulate
229	− 21.5	− 36.9	5.7	Hippocampus	Limbic	0.13	Hippocampus, hippocampal, learning task
96	29.3	− 19.6	− 26.3	Parahippocampal	Limbic	0.12	Medial temporal, hippocampus, parahippocampal

Bold values indicate the top two nodes that are shared across these phenotypes, and the remaining structures are largely implicated in learning and memory processes.

**Table 4:** Most important functional connectivity regions for worse memory and accelerated epigenetic age.

Node #	X	Y	Z	Region	Lobe	Centrality	Top neurosynth terms
Worse memory							
** 59**	**43.4**	** − 26.5**	** − 24.6**	**InfTempGyrus**	**Temporal**	**0.19**	**Photographs, temporal lobe, medial temporal**
** 234**	** − 30.5**	** − 23.9**	** − 26.6**	**Parahipp**	**Limbic**	**0.19**	**Lobe mtl, hippocampal, mtl**
122	13.7	− 4.2	20.9	Caudate	Subcortical	0.14	Caudate, caudate nucleus, nucleus
58	40.3	− 11.3	− 35.8	InfTempGyrus	Motor	0.13	Face recognition, medial temporal, temporal lobe
217	− 23.6	− 41.3	19.9	NA	Limbic	0.13	N/A
230	− 32.1	− 40.2	− 4	Hippocampus	Limbic	0.13	Hippocampal, medial temporal, hippocampus
More age accelerated							
** 59**	**43.4**	** − 26.5**	** − 24.6**	**InfTempGyrus**	**Temporal**	**0.21**	**Photographs, temporal lobe, medial temporal**
2	9.6	17.8	− 19.5	OrbFrontal	Prefrontal	0.15	Interpersonal, frontotemporal, cognitive emotional
120	21.2	− 36.4	22.6	NA	Subcortical	0.15	N/A
92	31.2	3.7	− 21.6	Amygdala	Limbic	0.14	Amygdala, amygdala insula, fear
** 234**	** − 30.5**	** − 23.9**	** − 26.6**	**Parahipp**	**Limbic**	**0.14**	**Lobe mtl, hippocampal, mtl**
127	12.3	− 27.7	13.5	Thalamus	Subcortical	0.13	Caudate nucleus, thalamic, insula inferior

Bold values indicate the top nodes (as well as 2nd memory node and 4th AgeAccelGrim node) that are shared across these phenotypes, and the remaining cortical structures are largely implicated in learning and memory processes.

## Discussion

Our findings provide evidence that epigenetic age acceleration is associated with differences in cognitive abilities for both fluid and crystallized intelligence, and that functional connectivity profiles that predict AgeAccelGrim are similar to functional connectivity profiles that predict memory abilities. Specifically, individuals with more epigenetic age acceleration tended to perform worse on tasks that spanned a wide variety of cognitive faculties, and brain regions crucial for successful memory formation were most important for predicting AgeAccelGrim in the aging brain. These differences cannot be explained by chronological age alone, in that AgeAccelGrim and chronological age are orthogonal variables, and both were included/controlled for in each statistical model. This suggests that epigenetic age explains a unique portion of variance of cognitive ability that chronological age does not capture, and this relationship may be explained by connectivity of memory brain structures primarily within the limbic system. Furthermore, at the behavioral level the effect of epigenetic age on cognition seems to negatively correlate with multiple domains and is not limited to fluid intelligence metrics. Interestingly the estimated slope values for AgeAccelGrim and chronological age were relatively close in standardized value across the domains, which may suggest that both factors are similarly important for predicting cognitive performance.

While AgeAccelGrim was associated with all cognitive factors, evidence from the brain using state-of-the-art rCPM functional connectivity analysis suggested that memory-related cognitive processes shared information with functional connectomes predicting AgeAccelGrim. This was reflected in the brain systems identified as important using rCPM, which largely implicated sub-cortical structures in the medial temporal lobe and limbic system. Connectivity within these nearby structures has been previously shown to change through aging and relates to cognitive outcomes^57–59^. Furthermore, the direction of connectivity (positive vs. negative) depends on the exact structures measured in these systems, such as the head, tail, or body of the hippocampus^60^. Model systems work corroborates with human studies, such that differential connectivity in CA1 and CA3 regions of the hippocampus in rats undergoing resting-state fMRI is associated with age-related memory deficits^61^. Taken together, these brain structures are known to be important for aging and cognitive change, and here we demonstrate that epigenetic age acceleration captures a unique portion of this functional brain organization as it pertains to these differences.

There are several limitations to the current research. Ideally, epigenetic age indices are tracked longitudinally in temporal synchrony with cognitive measurements and fMRI to assess within-person dynamics of these three factors through time. Access to this data could facilitate answering the question if epigenetic age acceleration precedes or follows cognitive decline within an individual as they age. While this would not be a fully causal explanation, it would provide insight into understanding which of these factors is first impacted by the aging process. We hypothesize that epigenetic age acceleration does in fact precede deficits in cognitive performance and differential functional connectivity, but more studies would need to be designed to provide evidence for an answer to that question. Another potential limitation of this study is the focus on one specific epigenetic clock. Our specific interest in using AgeAccelGrim for hypothesis testing on cognitive and functional brain differences was developed apriori. However, we have included results for both Horvath’s clock and PhenoAge in Figs. S5, S6 and Table S2, which show that these two clocks are in fact also negatively associated with cognition, but not above and beyond the effects of chronological age and AgeAccelGrim. We are not claiming that AgeAccelGrim is the only clock to investigate in this research context, and acknowledge that further advancements in the future will be developed that improve upon the innovations of AgeAccelGrim. Another limitation of this study is the participant sample, which consists of a relatively small sample of healthy older adults. The population sample for the original GrimAge work consisted of 50% White/Caucasian, 40% African American, and 10% Hispanic^15^. The majority of our studied sample was White/Caucasian, which is a potential limitation for the current research. Estimating the average association between epigenetic age acceleration, cognition, and functional brain connectivity would require a larger and more racially diverse sample from across the lifespan, as well as across the entire spectrum of general health. An important avenue for future research would be to determine if the relationship between epigenetic age acceleration, cognition, and functional brain connectivity would be heightened or diminished if a sample fully representative of the population with respect to health and race were assessed.

Future work should incorporate epigenetic and neuroimaging data in a prospective model to assess how pathological cognitive aging diverges from healthy cognitive aging. This could manifest as mild cognitive impairment (MCI) or more severe cases such as Alzheimer’s disease and Alzheimer’s disease related dementias (AD/ADRD). A future goal is to be able to estimate the likelihood of healthy and pathological development using epigenetic age, so that we can build better systems for predictive diagnostics and early intervention for specific persons. This could be accomplished through a rich and interdisciplinary individual differences approach to studying cognitive aging, by tracking epigenomic, phenotypic, and neuroimaging data together through time longitudinally. While further research is necessary for direct applications of these findings, epigenetic age acceleration together with neuroimaging in the future could be used as an important factor to consider for assessing risk of cognitive decline for individuals. The current research provides evidence that this could be a promising future approach for prospectively predicting cognitive decline risk, as the collection of epigenetic age and brain data becomes less expensive, and the quantification of this specific risk is analyzed with a sample representative of the general population In the future and following more research on this topic, these epigenetic age and neuroimaging measurements could be used as tools for diagnostics toward potential intervention, to help prevent cognitive decline for individuals at high risk who have yet to show symptoms. In summary, this research provides further evidence for the important role of epigenetic aging processes on the development and maintenance of healthy cognitive capacities, and suggests that functional connectivity within relevant brain structures for these capacities is differentially modulated through epigenetic age.

## Methods

### Participant sample

Protocols for data collection and analysis were evaluated and approved by the University of Virginia Health Sciences Institutional Review Board (Protocol HS19498). All participants provided written informed consent before participation. Participants underwent a 60-min scan protocol that consisted of functional and structural acquisition sequences. Participants were then led to a clinic where blood was drawn from a phlebotomist for downstream epigenetic analysis. No clinical data was collected from participants. The cognitive data collection, typically happened on a different day due to time constraints, and is described below.

The VCAP cognitive battery was measured longitudinally, across short and long time-scales using a variable retest interval and measurement burst design. For each occasion, participants’ cognitive abilities were surveyed three times in two-hour sessions, with each session being separated by two weeks. For the current research, we are only examining the occasion (three sessions per participant, *N* = 103) that is closest in time to the provided epigenetic sample and fMRI scan. Only 98 out of the 103 participants underwent fMRI, thus the neuroimaging analysis and results are based on the 98 participants who completed our fMRI protocol, while the behavioral results include all 103 participants. 73 females and 30 males with an average age of 68.98 years completed the study with the following race distribution: 90% Caucasian, 6% African American, 3% Native American, 1% Asian.

### Cognitive measurements

Each cognitive scale name has an associated abbreviation in parentheses for its name which is used in all figures and tables in order to conserve space. Descriptions of these tasks were abbreviated from descriptions previously published^8^. Sum scores of each cognitive task were computed as the basis for the bootEGA model.

#### Processing speed

The three instruments used to assess processing speed were Digit Symbol Substitution (DigSym), Pattern Comparison (PatCom), and Letter Comparison (LetCom). DigSym involves referencing a code table to write symbols arbitrarily paired with digits as quickly as possible^62^. PatCom involves determining if two line patterns are the same or different as quickly as possible^63^. LetCom involves determining if two letter strings are the same or different as quickly as possible^63^.

#### Memory

The three instruments used to assess memory were Word Recall (Recall), Logical Memory (LogMem), and Paired Associates (PAssoc). Recall requires listening to a list of 12 unrelated words and then immediately recalling as many as possible^64^. LogMem requires listening to a story and then immediately recalling as many details of that story as possible^64^. PAssoc requires listening to six pairs of unrelated words, and then recalling the second member of the pair after being cued by the first member^65^.

#### Spatial visualization

The three instruments used to assess spatial visualization were Spatial Relations (SpaRel), Paper Folding (PapFld), and Form Boards (FrmBrd). SpaRel involves identifying which three-dimensional figures corresponds to which two-dimensional figure if it were assembled^66^. PapFld involves selecting the pattern of holes that would result if a piece of paper were to be folded and a hole were punched in the specified location^67^. FrmBrd involves selecting pieces that could be constructed to fill a designated form^67^.

#### Reasoning

The three instruments used to assess reasoning were Matrix Reasoning (MatRea), Shipley Abstraction (Ship), and Letter Sets (LetSet). MatRea requires choosing a solution from alternatives to complete the missing cell in a matrix of geometric patterns^68^. Ship requires determining the best option for completing a pattern from a series of elements^69^. LetSet requires identifying the outlier among sets of letters that does not follow the same pattern/ rule as the others^67^.

#### Vocabulary

The three instruments used to assess vocabulary were WAIS Vocabulary (Vocab), Synonym Vocabulary (SynVoc), and Antonym Vocabulary (AntVoc). Vocab asks participants to provide a definition for each word where scores are either incorrect, incomplete, partially correct, or correct^62^. SynVoc asks participants to identify which of five words is the synonym to the target word^70^. AntVoc asks participants to identify which of five words is the antonym to the target word^70^.

### Epigenetic age

Eight and a half milliliters of whole blood were drawn into a PAXgene Blood DNA Tube (PreAnalytiX, Hombrechtikon, Switzerland). Samples were stored at 20 °C for short-term storage (up to 3 months) then transferred to − 80 °C for long-term storage. DNA was extracted using the PAXgene Blood DNA kit (PreAnalytiX, Hombrechtikon, Switzerland) according to manufacturer instructions. DNA concentration was determined by Quant-iT™ PicoGreen^®^ dsDNA reagent (Thermofisher Scientific, Waltham, MA, USA) per manufacturers instruction. Florescence was detected using a Tecan Infinite M200 Pro microplate reader (Tecan, Switzerland). 500 ng of DNA was bisulfite treated using a Zymo EZ DNA Methylation kit (Zymo Research, Irvine, CA) using PCR conditions for Illumina's Infinium Methylation assay (95 °C for 30 s, 50 °C for 60 min × 16 cycles). DNA methylation was assayed using the Illumina Infinium MethylationEPIC BeadChips. Briefly, a total of 4μL of bisulfite converted DNA was hybridized to Illumina BeadChips using the manufacturer’s protocols. Samples were denatured and amplified overnight for 20 to 24 h. Fragmentation, precipitation, and resuspension of the samples followed overnight incubation, before hybridization to EPIC BeadChips for 16 to 24 h. BeadChips were then washed to remove any unhybridized DNA and labeled with nucleotides to extend the primers to the DNA sample. Following the Infinium HD Methylation protocol, the BeadChips were imaged using the Illumina iScan system (Illumina).

Raw .idat files were read and preprocessed using the *minfi* package in R^71,72^. The data set was preprocessed using noob for background subtraction and dye-bias normalization. All methylation values with detection P > 0.01 were set to missing (median sample: 765 probes, range: 319 to 4453), and probes with > 1% missing values (*n* = 6663) were removed from further analysis. All samples were checked and confirmed to ensure that predicted sex matched reported sex. Additionally, samples were checked for excessive missing data (> 5%) and unusual cell mixture estimates, which was estimated using the Houseman method as implemented in *minfi*^73,74^. All samples passed these quality controls. Principal components analysis, as implemented in the *shinyMethyl* package in R, was used to examine batch effects^75^. The first seven principal components were examined using plots and potential batch effects were tested using linear models. Principal components 3 and 6, which accounted for 2.38% and 1.65% of total variance respectively, were associated with position on the array (PC3: *F*_(7, 100)_ = 6.668, *p* = 1.77e–6, adjusted *R*^2^ = 0.271; PC6: *F*_(7, 100)_ = 2.328, *p* = 0.030, adjusted *R*^2^ = 0.080). Principal components 1, 4, and 5, which accounted for 3.63%, 1.89%, and 1.77% of the total variance were associated with bisulfite conversion plate (PC1: *F*_(1, 106)_ = 9.918, *p* = 0.002, adjusted *R*^2^ = 0.077; PC4: *F*_(1, 100)_ = 34.04, *p* = 5.932e–8, adjusted *R*^2^ = 0.236; PC5: *F*_(1, 100)_ = 31.07, *p* = 1.91e–7, adjusted *R*^2^ = 0.219). Principal components 4 and 5 were associated with array (PC4: *F*_(13, 94)_ = 4.332, *p* = 1.14e–5, adjusted *R*^2^ = 0.288; PC5: *F*_(13, 94)_ = 4.229, *p* = 1.06e–5, adjusted *R*^2^ = 0.282). Bisulfite conversion plate and array number were associated with each other, as samples on the same array originated from the same bisulfite conversion plate. Because samples were randomized across plates and arrays, and proportions of variance explained by associated principal components were low, no batch correction method was used. The *ewastools* package in R was used to assess Illumina quality control metrics and call genotypes and donor IDs to ensure the identity of repeated samples from the same individual^76^. All samples passed Illumina quality controls.

To determine assay variability, we included one set of five technical replicates and an additional three sets of two technical replicates. After quality control filters and normalization procedures were applied, the 5000 CpGs with the most variable M values were used as input for calculating Pearson’s correlation coefficients among all pairwise combinations of samples. Pearson’s correlation of unrelated samples (different individuals) were below 0.8, while correlations of technical replicates ranged from 0.988 to 0.994, indicating high agreement between technical replicates.

Unnormalized betas were filtered to include CpGs specified by Horvath as necessary for calculation of various clocks. The betas were uploaded to Horvath’s online DNA methylation age calculator (https://dnamage.genetics.ucla.edu), which provides measures of Horvath’s multi-tissue age estimator, DNA methylation GrimAge, and cell type abundance^10,15^. A sample annotation file was included. The options to normalize data and apply advanced analysis were selected. Technical replicates were used to determine measurement error of DNAmAge, the output of Horvath’s multi-tissue age estimator. The absolute difference of DNAmAge between technical replicate pairs was taken, as was the highest absolute difference in the set of five technical replicates. The median of the absolute difference was 2.02 years (range: 0.44–5.73 years), comparable to previous reports of measurement error being approximately 2.41 years^77^.

### Functional magnetic resonance imaging

#### Imaging parameters and acquisition

MRI scanning was performed at the University of Virginia Fontaine Research Park on a Siemens 3 Tesla MAGNETOM Prisma Fit high-speed imaging device equipped with a 32-channel head-coil. First, high-resolution T1-weighted anatomical images were acquired using Siemens' magnetization-prepared rapid-acquired gradient echo (MPRAGE) pulse sequence with the following specifications: echo time (TE) = 2.98 ms; repetition time (TR) = 2300 ms; flip angle (FA) = 9°; image matrix = 240 mm × 256 mm; slice thickness = 1 mm; 208 slices. Then, whole-brain functional images were acquired using a T2*-weighted echo planar imaging (EPI) sequence sensitive to BOLD contrast with the following specifications: TE = 30 ms; TR = 800 ms; FA = 52°; image matrix = 90 mm × 90 mm; slice thickness = 2.4 mm; slice gap = 2.4 mm; 660 slices. We collected two 610 volume resting-state functional runs, totaling 976 s of resting-state functional imaging data for each participant. A black crosshair on a gray background was presented using an LCD AVOTEC projector onto a screen located behind the participant’s head and viewed through an integrated head-coil mirror.

#### Pre-processing with *fMRIPrep*

Results included in this manuscript come from preprocessing performed using *fMRIPrep* 21.0.2 (RRID:SCR_016216)^78^, which is based on *Nipype* 1.6.1 (RRID:SCR_002502)^79^. Many internal operations of *fMRIPrep* use *Nilearn* 0.8.1^80^, mostly within the functional processing workflow. For more details of the pipeline, see the section corresponding to workflows in *fMRIPrep*’s documentation. For each participant, the T1-weighted (T1w) image was corrected for intensity non-uniformity (INU) with N4BiasFieldCorrection^81^, distributed with ANTs 2.3.3 (RRID:SCR_004757)^82^, and used as T1w-reference throughout the workflow. The T1w-reference was then skull-stripped with a *Nipype* implementation of the antsBrainExtraction.sh workflow (from ANTs), using OASIS30ANTs as target template. Brain tissue segmentation of cerebrospinal fluid (CSF), white-matter (WM) and gray-matter (GM) was performed on the brain-extracted T1w using fast (FSL 6.0.5.1:57b01774, RRID:SCR_002823)^83^. Volume-based spatial normalization to one standard space (MNI152NLin2009cAsym) was performed through nonlinear registration with antsRegistration (ANTs 2.3.3), using brain-extracted versions of both T1w reference and the T1w template. The following template was selected for spatial normalization: *ICBM 152 Nonlinear Asymmetrical template version 2009c* (RRID:SCR_008796; TemplateFlow ID: MNI152NLin2009cAsym)^84^. A deformation field to correct for susceptibility distortions was estimated based on *fMRIPrep*’s *fieldmap-less* approach. The deformation field is that resulting from co-registering the EPI reference to the same-subject T1w-reference with its intensity inverted^85,86^. Registration is performed with antsRegistration (ANTs 2.3.3), and the process regularized by constraining deformation to be nonzero only along the phase-encoding direction, and modulated with an average fieldmap template^87^.

For each of the two BOLD resting-state runs per subject, the following preprocessing was performed. First, a reference volume and its skull-stripped version were generated using a custom methodology of *fMRIPrep*. Head-motion parameters with respect to the BOLD reference (transformation matrices, and six corresponding rotation and translation parameters) were estimated before any spatiotemporal filtering using mcflirt (FSL 6.0.5.1:57b01774)^88^. The estimated *fieldmap* was then aligned with rigid-registration to the target EPI (echo-planar imaging) reference run. The field coefficients were mapped on to the reference EPI using the transform. BOLD runs were slice-time corrected to 0.351 s (0.5 of slice acquisition range 0–0.703 s) using 3dTshift from AFNI (RRID:SCR_005927)^89^. The BOLD reference was then co-registered to the T1w reference using mri_coreg (FreeSurfer) followed by flirt (FSL 6.0.5.1:57b01774)^90^ with the boundary-based registration cost-function^91^. Co-registration was configured with six degrees of freedom.

Several confounding time-series were calculated based on the *preprocessed BOLD*: framewise displacement (FD), DVARS and three region-wise global signals. FD was computed using two formulations following Power (absolute sum of relative motions^92^) and Jenkinson (relative root mean square displacement between affines^88^). FD and DVARS were calculated for each functional run, both using their implementations in *Nipype*^92^. The three global signals were extracted within the CSF, the WM, and the whole-brain masks. Additionally, a set of physiological regressors were extracted to allow for component-based noise correction (*CompCor*)^93^. Principal components were estimated after high-pass filtering the *preprocessed BOLD* time-series (using a discrete cosine filter with 128 s cut-off) for the two *CompCor* variants: temporal (tCompCor) and anatomical (aCompCor). tCompCor components were then calculated from the top 2% variable voxels within the brain mask. For aCompCor, three probabilistic masks (CSF, WM and combined CSF + WM) were generated in anatomical space. The implementation differs^93^ in that instead of eroding the masks by 2 pixels on BOLD space, the aCompCor masks are subtracted a mask of pixels that likely contain a volume fraction of GM. This mask is obtained by thresholding the corresponding partial volume map at 0.05, and it ensures components are not extracted from voxels containing a minimal fraction of GM. Finally, these masks are resampled into BOLD space and binarized by thresholding at 0.99 (as in the original implementation). Components are also calculated separately within the WM and CSF masks. For each CompCor decomposition, the *k* components with the largest singular values are retained, such that the retained components’ time series are sufficient to explain 50 percent of variance across the nuisance mask (CSF, WM, combined, or temporal). The remaining components are dropped from consideration. The head-motion estimates calculated in the correction step were also placed within the corresponding confounds file. The confound time series derived from head motion estimates and global signals were expanded with the inclusion of temporal derivatives and quadratic terms for each^94^. Frames that exceeded a threshold of 0.5 mm FD or 1.5 standardized DVARS were annotated as motion outliers.

The BOLD time-series were resampled into standard space, generating a *preprocessed BOLD run in MNI152NLin2009cAsym space*. All resamplings can be performed with *a single interpolation step* by composing all the pertinent transformations (i.e. head-motion transform matrices, susceptibility distortion correction when available, and co-registrations to anatomical and output spaces). Gridded (volumetric) resamplings were performed using antsApplyTransforms (ANTs), configured with Lanczos interpolation to minimize the smoothing effects of other kernels^95^.

#### Parcellation and image preparation

At the parcellation step, functional images were high-pass filtered at 0.008 Hz and cleaned with the following fMRIPrep confound derivatives to account for global BOLD signal outside of gray matter (csf, white_matter), primary data-driven estimated noise components (tcompcor, a_comp_cor_00, a_comp_cor_01) and motion-related parameters (trans_x, trans_x_power2, trans_y, trans_y_power2, trans_z, trans_z_power2, rot_x, rot_x_power2, rot_y, rot_y_power2, rot_z, rot_z_power2). For both resting-state functional scans, parcellation was performed by taking the framewise average of the voxel-wise signals in each of the 268 nodes from the Shen atlas^96^. The Shen atlas is a functionally defined parcellation that covers the whole brain, including cortex, subcortex, and cerebellum. The two resting-state scans were concatenated along the time dimension before calculating connectivity, as this can improve reliability of estimates^97^. We calculated Fisher Z transformed Pearson correlation coefficients between the activity time courses of all possible pairs of nodes to construct 268 × 268 symmetric functional connectivity matrices. Only the lower triangle of each functional connectivity matrix was extracted and vectorized, discarding the constant diagonal, resulting in 35,778 unique connections/edges, which served as input features (i.e. columns in the design matrix) to rCPM.

### Statistical analysis

#### Bootstrap exploratory graph analysis

In order to reduce the dimensionality of the cognitive measurements into a latent space, we applied bootEGA using the *EGAnet* package in R^98^. bootEGA is a community detection and network analysis method to evaluate the dimensional structure estimated using Exploratory Graph Analysis (EGA)^99,100^. The general approach of bootEGA is to generate bootstrap samples and apply EGA to each replicate sample, forming a sampling distribution of EGA results. EGA models a collection of variables through estimation of a sparse regularized partial correlation matrix using the graphical LASSO (GLASSO) procedure^101^.

The parametric bootstrap procedure begins by estimating a network using EGA and then generating new replicate data from a multivariate normal distribution (with the same number of cases as the original data). EGA is then applied to the replicate data, continuing iteratively until the desired number of samples is achieved (1000 iterations used). The result is a sampling distribution of EGA networks. From this sampling distribution, a median (or typical) network structure was estimated by computing the median value of each edge across the replicate networks, resulting in a single network. Such a network represents the “typical” network structure of the sampling distribution. The Louvain community detection algorithm was then applied, resulting in dimensions that would be expected for a typical network from the EGA sampling distribution (identical community membership estimated by the Walktrap algorithm for comparison)^102^. One metric for structural consistency is *item stability* or the robustness of each item’s placement within each empirically derived dimension. Item stability is estimated by computing the proportion of times each item is placed in each dimension. This metric provides information about which items are leading to structural consistency (replicating often in their empirically derived dimension) or inconsistency (replicating often in other dimensions).

We computed standardized network community scores for each community from the model-implied graph from EGA. The standardized network community scores are linear combinations of the original measurements within the cognitive battery. These network scores are compressed representations of the original data and are analogous to component vectors from principal components analysis or factor scores from factor analysis^103^. Each cognitive dimension derived from EGA (four, in this case) has its own network score vector. This means each participant has four network scores for each session, and each network score is computed as a linear combination of the weighted items that load onto each community.

**Figure 6:**
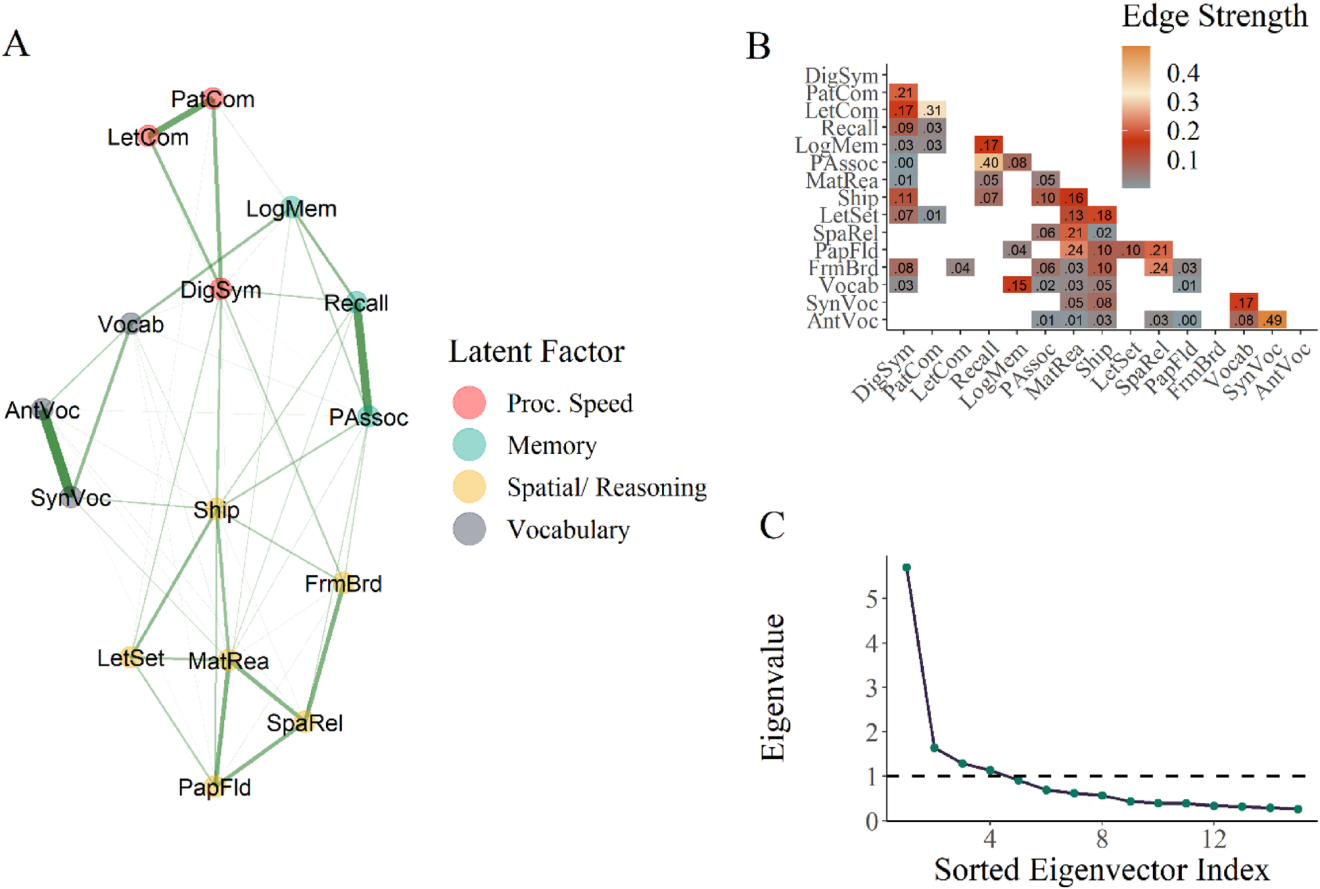
Four latent domains estimated from the cognitive battery using bootEGA. Panel (**A**) shows the estimated graph of the cognitive battery, where distinct colors reflect distinct communities and the size of the edges between nodes reflects the strength of the connection. Panel (**B**) shows the regularized partial correlation matrix that is a numeric representation of the graph, where white space indicates no connection between those nodes, and the cell entries represent edge strength between each node. This is the adjacency matrix for the graph and was estimated using GLASSO. Panel (**C**) shows the eigenvalue–eigenvector decomposition of the cognitive battery, providing additional supporting evidence that there are approximately 4 latent variables among the original set of items.

The results from Bootstrap Exploratory Graph Analysis (bootEGA) suggest four distinct communities, which roughly map to the cognitive domains of processing speed, memory, reasoning/ spatial visualization, and vocabulary (see Fig. 6). These latent variable communities were stably estimated, such that items consistently loaded into the same factor across repeated iterations (for metrics that demonstrate high stability, please see Fig. S4). The dimensionality assessment of four communities from bootEGA corroborates with the Scree plot in Fig. 6 using eigenvalue–eigenvector decomposition. The number of eigenvalues greater than 1 is a rough heuristic estimate of the number of distinct communities contained in a collection of variables (in this case, 4). The likely reason for the reasoning and spatial visualization factors to be collapsed into the same community is the inclusion of the matrix reasoning item, which loads most strongly onto this latent variable and requires spatial abilities to solve appropriately. It is also common to find that reasoning and spatial visualization abilities are highly positively correlated with each other^104^. It is important to note that the four derived latent factors are not orthogonal, and items from different communities can have non-zero weights onto other communities. This makes sense to allow from a cognitive perspective, as we do not expect any of these domains to be fully independent of each other.

#### Hierarchical Bayesian model approach

In order to model cognitive performance as a function of AgeAccelGrim, chronological age, sex, and blood cell count covariates (abbreviated as NK, Mono, Gran, PlasmaBlast, CD8pCD28nCD45Ran, CD8.naive, CD4.naive), we fit a multivariate hierarchical Bayesian generalized linear model with a Student-T likelihood. Because blood cell counts are known to influence DNAmGrimAge, we account for these age-related changes in immune cell populations to ensure the epigenetic age acceleration parameters are not merely indicative of blood cell counts^105^. We used the Student-T likelihood as an alternative to the traditional Gaussian likelihood, because the Student-T alternative is more robust to outliers and converges to the Gaussian solution when Gaussian assumptions are in fact met^106^. Interpreting the coefficients of Student-T regression (also known as robust regression in a Bayesian framework) is similar to traditional regression with a Gaussian likelihood. Each latent cognitive domain response variable was jointly estimated in a multivariate model to account for correlation across each domain.

We used the *brms* software package in R and the state-of-the-art Hamiltonian Monte-Carlo No-U-Turn sampler (NUTS) for Bayesian computation and inference^107,108^. Default priors on the intercepts were generated by Student-T (ν = 3, μ = 0.2, σ = 2.5), and the σ scale parameters were generated by Student-T (ν = 3, μ = 0, σ = 2.5). Priors for the ν degrees of freedom parameter were generated by γ (α = 2, β = 0.1), with special treatment of the vocabulary prior γ (α = 12, β = 0.1), due to its consistent underestimation of ν which would lead to unstable posteriors without being addressed. Priors for the correlation matrices between fixed and random effects were generated by LKJ-Cholesky (η = 1). We ran 7 independent Markov chains each with 20,000 total iterations, including 10,000 warm-up iterations. We fixed the target average proposal acceptance probability to 99% to improve the quality of sampling and thus the resulting posterior distributions. Convergence of the posteriors were confirmed with all R̂ ≈ 1.0, which assesses agreement across the Markov chains. Posterior predictive checks were used to assess model adequacy^109^.

Posterior distributions on the parameters can be inspected and inference can be employed using credible intervals (the Bayesian alternative to Frequentist confidence intervals). Additionally, we can simply look at the ratio of posterior density on respective sides of 0. Counting the number of observations in the sampled posterior distribution for a given parameter on the side of 0 that has the minority, and then multiplying that count by 2, results in a Bayesian analogue to a two-tailed Frequentist *p*-value. The *hypothesis* function in *brms* returns these posterior probabilities for inference^107^. This procedure is not directly sensitive to the prior relative to alternative procedures such as bayes factors, making it a more attractive option when the priors are used simply for regularization and are not informed by domain knowledge^110^.

There are several motivating factors for using Bayesian techniques for the current research. First, we can apply regularization priors to the regression coefficients as a way to employ shrinkage and mitigate potential for over-fitting, generated by β ~ N (μ = 0, σ = 10). Second, we can easily model hierarchy through probability distributions which allows for accurate estimation of subject-specific parameters, which we used to estimate intercepts and slopes through session to account for potential practice-effect noise. Third, we have explicit control over the probability distribution of the likelihood. This allows us to use more robust techniques such as Student-T regression. Overall, these three factors generally lead to more conservative and robust inference relative to the Frequentist approach^111^. Last, we can fit this hierarchical model in a multivariate context, which typically requires Bayesian sampling-based approaches rather than optimization-based techniques given there is scarce software support or implementation applying this specific Frequentist optimization problem.

#### Ridge regression connectome-based predictive modeling

It has been shown that treating connectivity vectors as columns for predicting behavior using ridge regression tends to perform better than other popular connectome-based modeling approaches^112^. Ridge regression is a linear supervised learning technique that regularizes model coefficients toward 0 with the canonical L2 norm. The regularization degree is governed by a single parameter, λ, where large values perform more shrinkage and small values perform less shrinkage. We performed rCPM using a repeated (*N* = 100) outer *K*-fold (*K* = 10) cross-validation procedure where individuals were split into 10 folds, models were trained using 9 of the folds, and then evaluated on the held-out fold. Within each cross-validation split, we tuned λ with an inner twofold cross-validation loop to conservatively estimate optimal regularization strength and overall prediction fit. The phenotypic outcomes were residualized with respect to confound/ nuisance variables specific for each analysis.

Prediction performance for the rCPM models were evaluated using the Spearman correlation, since successful rank prediction across participants was considered most important. To assess the statistical significance of prediction performance, we generated null distributions of expected performance metrics due to chance by permuting behavioral scores with respect to individuals and ran the rCPM pipeline for 1000 iterations. Then, we calculated a non-parametric *p*-value, which tallies the number of times the performance metric for each of the 1000 iterations of the null distribution exceeds the median performance metric of the 100 true iterations. The coefficient matrices were treated as networks to estimate importance of specific brain regions by calculating eigenvector centrality on the ridge regression coefficient matrix averaged across cross-validation iterations and separated by positive and negative sign. The top 2% of coefficients for both the positive and negative sign were used for the network visualizations.

### Supplementary Information

Supplementary Information.

## Data Availability

Deidentified data is available upon request. Please contact Andrew J. Graves (ajg3eh@virginia.edu) for additional data and/ or code requests. Analysis code and pre-processed data are available on GitHub (https://github.com/andrew-graves/ageaccelgrim_cog_brain).
