# Supplementary material for: Accelerated epigenetic age is associated with whole-brain functional connectivity and impaired cognitive performance in older adults
Source: Sci Rep. 2024 Apr 26;14:9646. doi: 10.1038/s41598-024-60311-3 (PMC11053089; doi:10.1038/s41598-024-60311-3)
Supplement: Supplementary file 1 — Supplementary Information. [file 41598_2024_60311_MOESM1_ESM.docx]

Supplementary Materials for

**Accelerated epigenetic age is associated with whole-brain functional connectivity and impaired cognitive performance in older adults**

Andrew J. Graves, Joshua S. Danoff, Minah Kim, Samantha R. Brindley, Amalia M. Skyberg, Stephanie N. Giamberardino, Morgan E. Lynch, Brenda C. Straka, Travis S. Lillard, Simon G. Gregory, Jessica J. Connelly, James P. Morris

*Corresponding author. Email: jpm5jb@virginia.edu

**This PDF file includes:**

Figs. S1 to S6

Tables S1 to S2


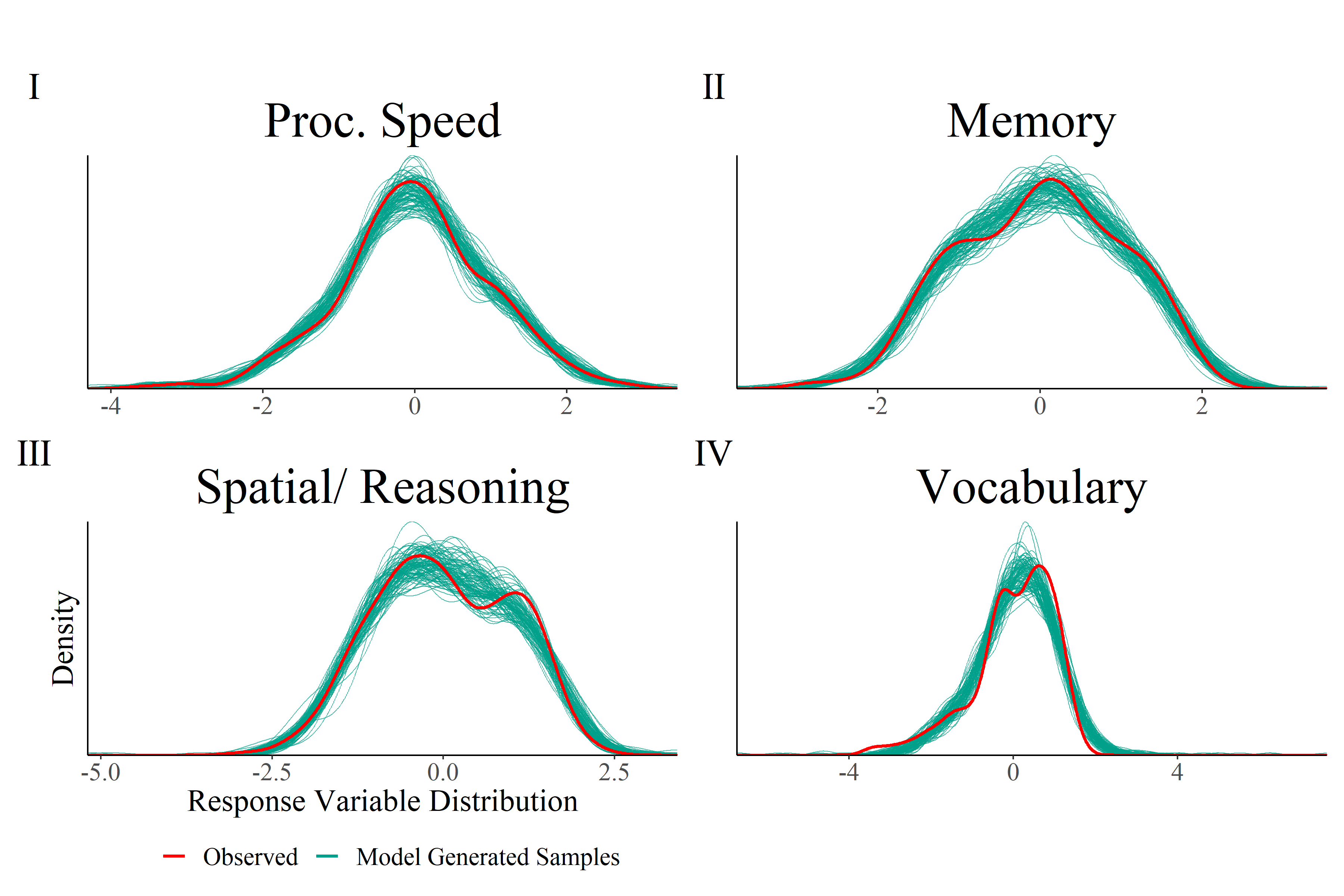
**Fig. S1. Posterior predictive distribution check shows good overlap with model simulations and observed data.** This provides visual diagnostic information that the Bayesian model is adequately specified, and that the mathematical assumptions appear sufficient for representing the data generation process. Each of these posterior distribution panels represent Student-T likelihoods jointly estimated from the four response variables in the multivariate hierarchical model. The Vocabulary response variable exhibits wider tails because the posterior estimation of the degrees of freedom was lower than the other three response variables, which motivated the use of a stronger prior specifically on the degrees of freedom parameter, υ, for Vocabulary. This improved model stability without sacrificing the capacity to do inference for this response variable.

**
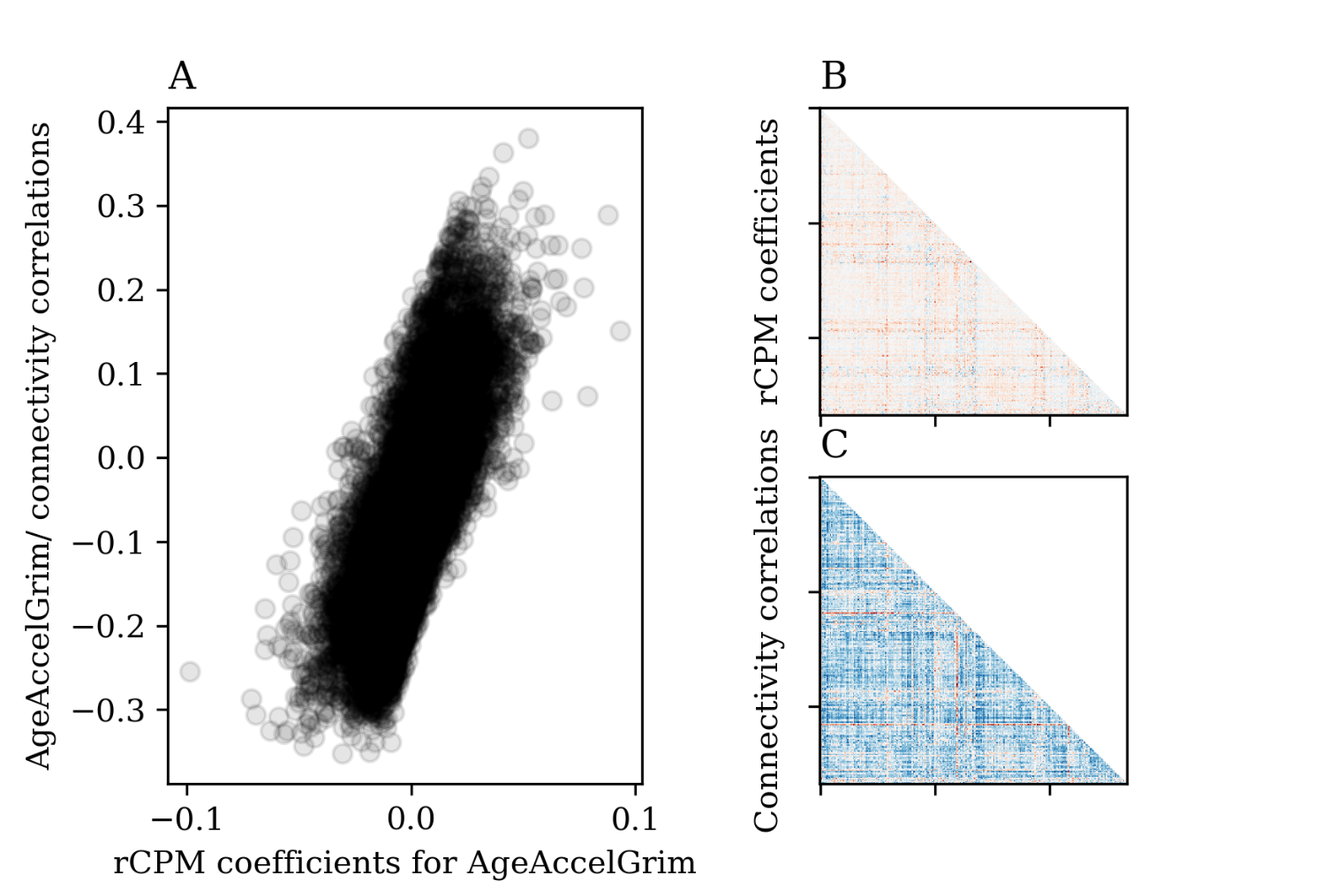
**

**Fig. S2. rCPM captures and filters functional connectivity-phenotype relationships.** Using AgeAccelGrim as a demonstrative example, the correlation between rCPM coefficients and simple bivariate correlations with phenotype is strong and positive. This is indicated both by the scatterplot in Panel A and the similarity between important nodes in the two matrix heatmaps in Panels B and C. Each row/column in the heatmap matrices corresponds to a unique brain region, and each cell entry represents the correlation between pairwise connectivity of those two regions and phenotype (i.e., AgeAccelGrim). This suggests that analyzing the degree network statistic of the rCPM coefficient matrix will recover which brain regions are uniquely important for predicting AgeAccelGrim, and can be interpreted in a similar fashion to summing the bivariate correlation values of a specific brain region to determine importance.

**
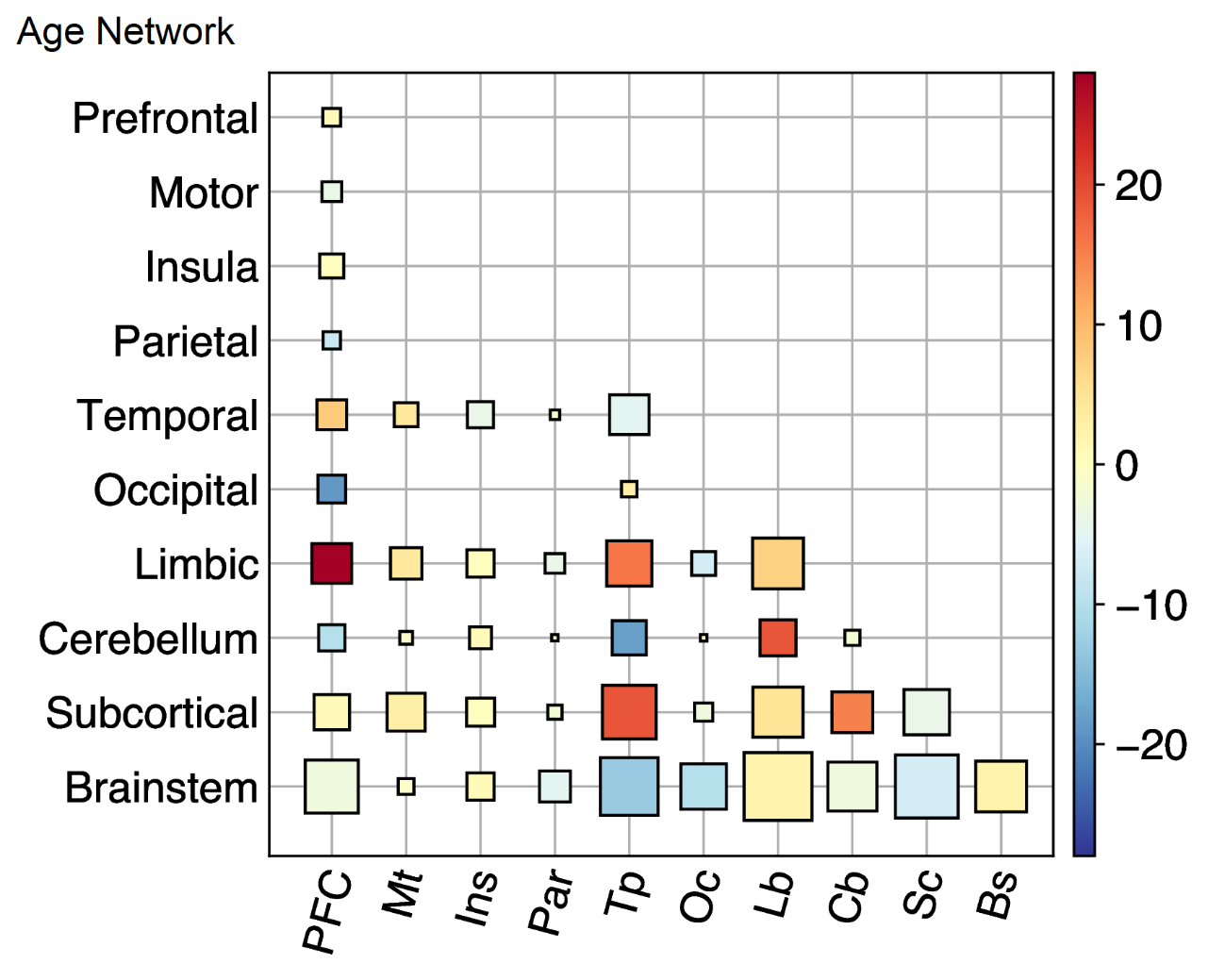
**

**Fig. S3. Networks reflective of age do not show opposite patterns in limbic system to memory networks.** Hinton plot visualizations show that memory (see Figure 5 in main manuscript) and age, in contrast to AgeAccelGrim, do not have opposing network structure patterns in the limbic system. Size corresponds to the sum of edges in the “high”- and “low” networks standardized by the number of possible edges between each pair of regions. Color corresponds to the difference between edges in the high- and low-phenotype networks, such that red corresponds to edges mostly in the older age network and blue corresponds to edges mostly in the younger age network.
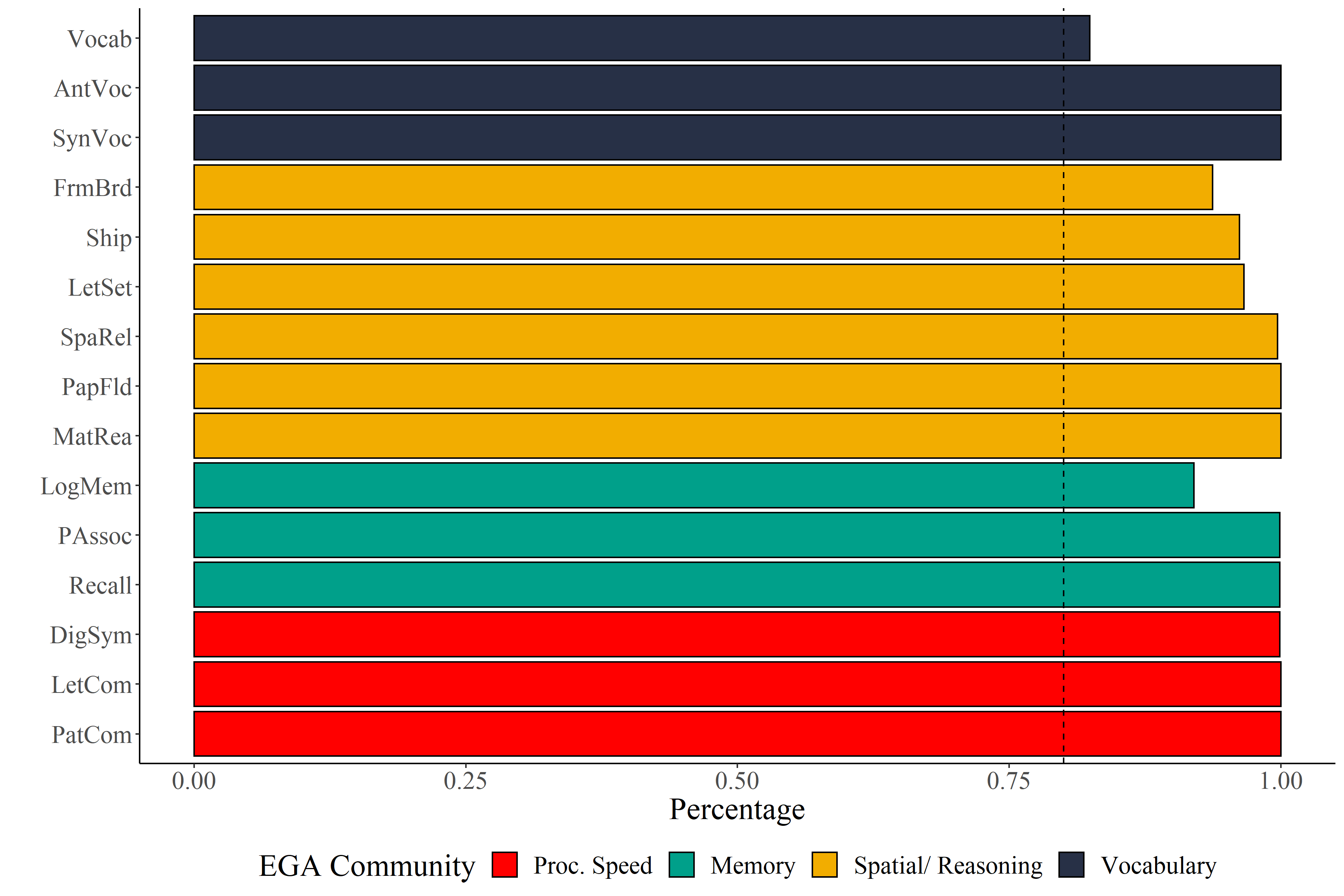


Fig. S4. All cognitive items were stably estimated into discrete communities from bootEGA. Stability was assessed via generation of 1000 multivariate normal replicates of the original data. Higher item percentages are roughly analogous to higher loadings in traditional factor analysis. The replications provide insight into the uncertainty of the community structure. Heuristically, items that demonstrate greater than 80% replication percentage are considered relatively stable, and each item exceeds that threshold. This suggests that this identical structure would likely replicate upon collection of a new sample.


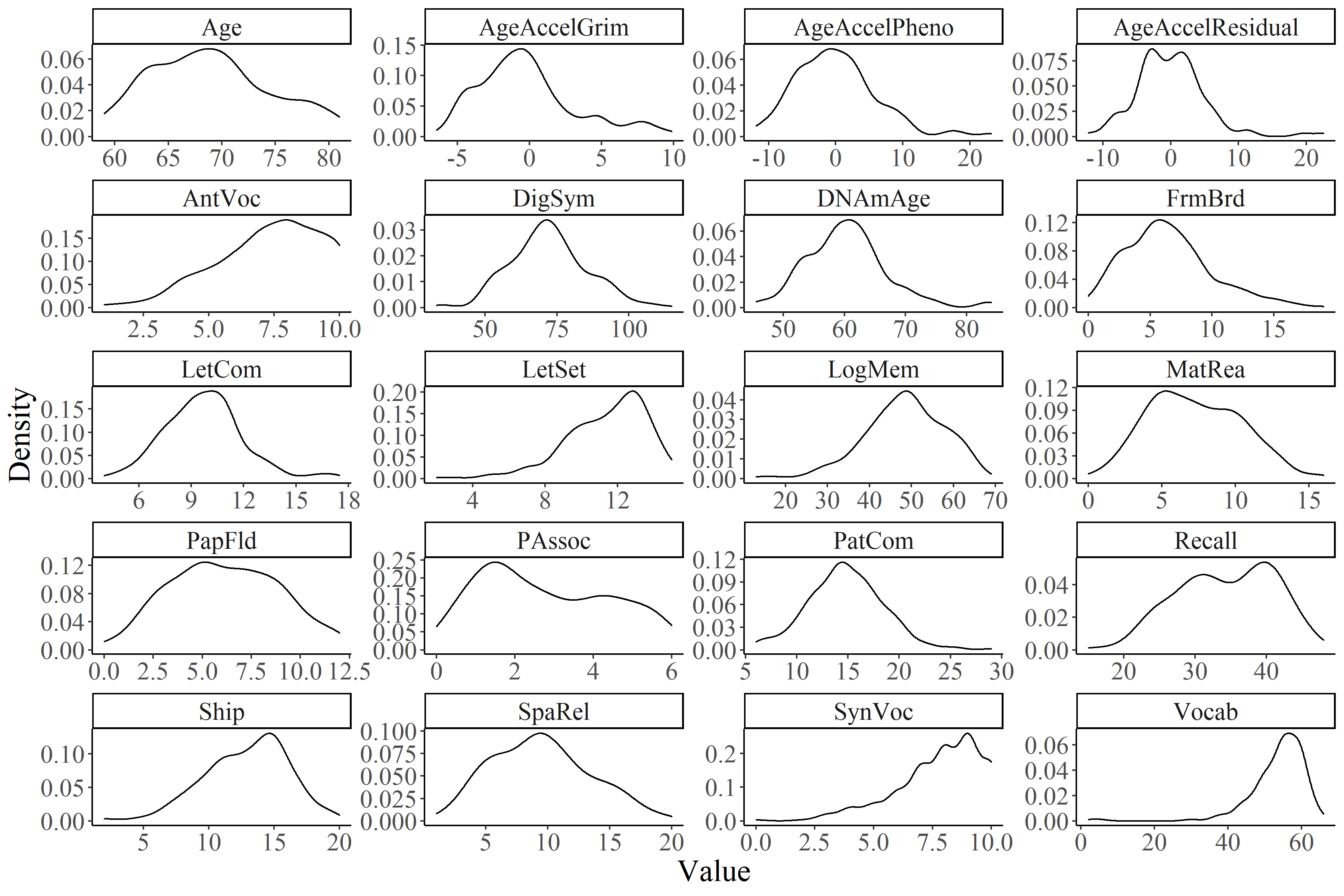


Fig. S5. Kernel density estimates for chronological age, epigenetic age acceleration values, and item-level cognitive assessments. This figure shows the shape and variability of the quantitative measurements from Table 1, as well as AgeAccelPheno and AgeAccelerationResidual for comparison.


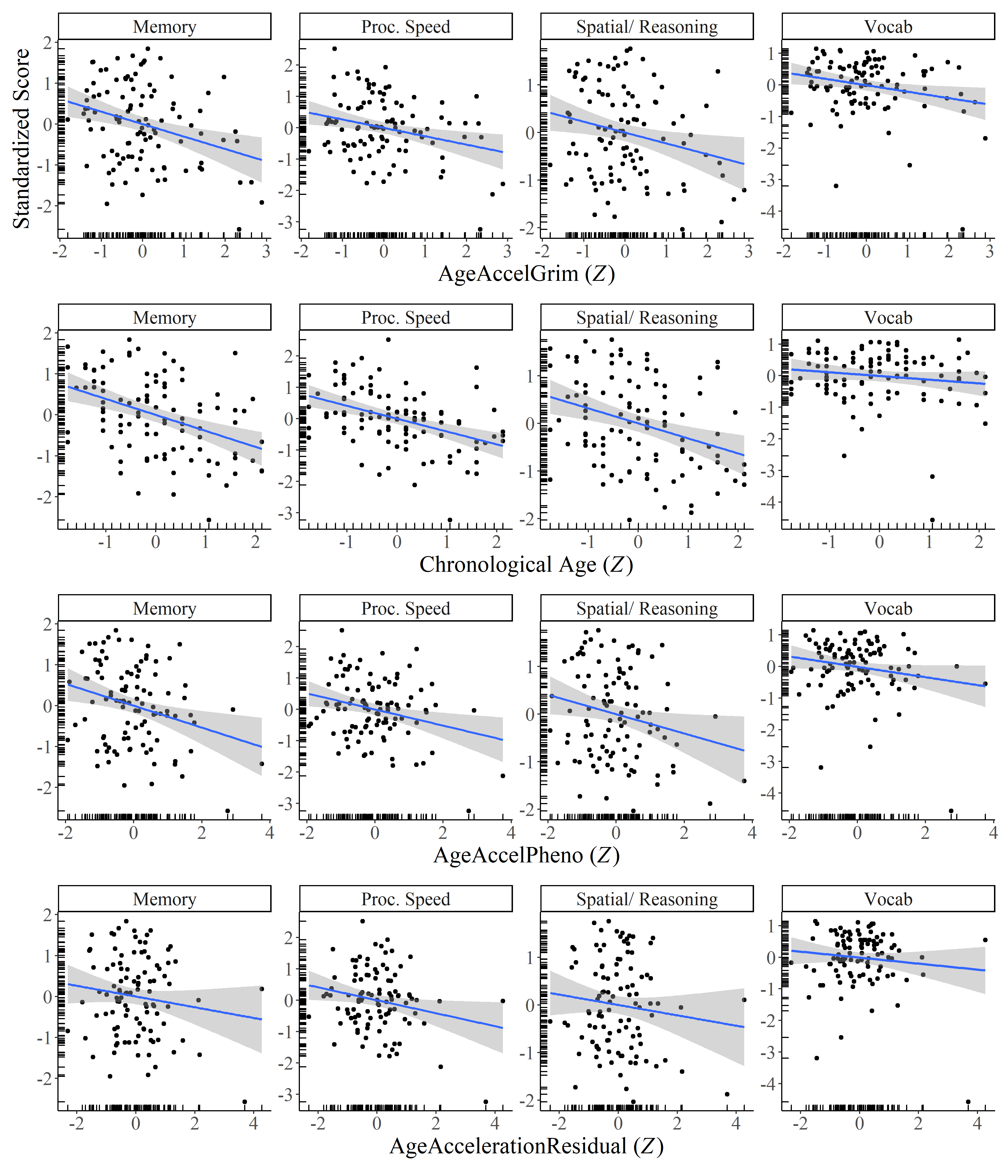
 Fig. S6. The relationship between chronological age, epigenetic age acceleration metrics, and cognition show similarly negative patterns. Rug-plots in the margins indicate the location of each measurement across each axis. Here, cognitive measurements are aggregated across session.

| *Node #* | *X* | *Y* | *Z* | *Region* | *Lobe* | *Centrality* | *Top Neurosynth Terms* |
| --- | --- | --- | --- | --- | --- | --- | --- |
| *Older Age* |  |  |  |  |  |  |  |
| 121 | 12.7 | 12.9 | 11.5 | Caudate | Subcortical | 0.14 | social, imagine, construction |
| 185 | -38 | 6.1 | -37.9 | Temporal Pole | Temporal | 0.14 | semantic memory, social interactions, default network |
| 194 | -49.3 | -4.7 | -37.4 | Inferior Temporal Gyrus | Temporal | 0.14 | caudate, caudate nucleus, nucleus |
| 87 | 28.4 | -53.8 | 7.1 | Ventral Posterior Cingulate | Limbic | 0.13 | N/A |
| 94 | 35.6 | -14.7 | -18.4 | Hippocampus | Limbic | 0.13 | hippocampus, hippocampal, memory |
| 202 | -30 | -5.8 | -40.9 | Parahippocampal | Temporal | 0.13 | progressive, aphasia, dementia |
| *Younger Age* |  |  |  |  |  |  |  |
| 132 | 6.3 | -24.9 | -17.5 | Brainstem | Brainstem | 0.17 | ventral tegmental, tegmental, midbrain |
| 195 | -37.8 | -13.2 | -29.3 | Inferior Temporal Gyrus | Temporal | 0.15 | hippocampus, medial temporal, hippocampal |
| 232 | -35.7 | -24.8 | -14.9 | Hippocampus | Limbic | 0.15 | anterior temporal, temporal, medial temporal |
| 135 | -18.2 | 19 | -21 | Orbitofrontal | Prefrontal | 0.14 | paralimbic, orbitofrontal, cortex amygdala |
| 136 | -5.8 | 18.2 | -21.6 | Orbitofrontal | Prefrontal | 0.14 | subgenual, major depression, depression |
| 120 | 21.2 | -36.4 | 22.6 | NA | Subcortical | 0.13 | N/A |

**Table S1. Most important functional connectivity regions for predicting older age and younger age.**

| *β coefficient* | *Proc. Speed* | *Memory* | *Spatial/ Reasoning* | *Vocabulary* |
| --- | --- | --- | --- | --- |
| *Parameters of Interest* | | | | |
| AgeAccelGrim | -0.21, *p* < .001 | -0.25, *p* < .001 | -0.19,  *p =* .003 | -0.15,  *p =* .027 |
| Age | -0.40,  *p* < .001 | -0.39,  *p* < .001 | -0.31,  *p <* .001 | -0.20, *p* < .001 |
| AgeAccelPheno | -0.08, *p =* .244 | -0.14, *p =* .042 | -0.10,  *p =* .178 | -0.14,  *p =* .073 |
| AgeAccelerationResidual | -0.07, *p =* .282 | 0.06,  *p =* .349 | 0.03,  *p =* .641 | 0.11,  *p* = .081 |

**Table S2. AgeAccelGrim negatively associates with cognition above and beyond AgeAccelPheno and AgeAccelerationResidual.** Four separate linear models of all cognitive domains indicate that AgeAccelGrim provides epigenetic aging information for predicting cognitive function unique from AgeAccelPheno and AgeAccelerationResidual. Note that AgeAccelPheno and AgeAccelerationResidual negatively correlate with many of the cognitive measurements above and beyond chronological age, but not when controlling for AgeAccelGrim.
